# Advanced methods of wearable ultrasound and photoacoustic imaging devices: A review

**DOI:** 10.1016/j.pacs.2026.100884

**Published:** 2026-09-15

**Authors:** Mingze Luo, Shunyao Zhang, Wentao Jiang, Jingyi Miao, Zihan Zhao, Lei S. Li

**Affiliations:** a Department of Electrical and Computer Engineering, Rice University, Houston, TX, 77005, United States; b Department of Bioengineering, Rice University, Houston, TX, 77005, United States

**Keywords:** Ultrasound Imaging, Photoacoustic Imaging, Wearable Devices, Wearable Patch, PMUT, CMUT, Spatial Encoding

## Abstract

Ultrasound imaging (USI) and photoacoustic imaging (PAI) are being adapted from console-based examinations to body-conformal formats for continuous and point-of-care monitoring. USI offers real-time anatomical and motion information, whereas PAI adds optical absorption contrast related to hemoglobin, oxygenation, temperature, and molecular composition. The challenge is no longer a single component. Wearable USI and PAI require soft mechanics, stable acoustic coupling, compact light delivery, low-power acquisition, safe operation, reliable data handling, and reconstruction methods that tolerate motion and imperfect contact. This review examines recent wearable USI and PAI technologies from device level to system level. We cover micromachined transducers, flexible and stretchable ultrasound patches, laser-diode and VCSEL-based PA excitation, compact PA detectors, multimodal PA/US integration, wireless electronics, spatial encoding, and reconstruction methods. Representative systems are compared in terms of form factor, imaging target, hardware architecture, and remaining bottlenecks, with emphasis on the gap between wearable probes and autonomous imaging platforms.

## 1 Introduction

Biomedical imaging is central to diagnosis, treatment guidance, and longitudinal health monitoring [[1], [2], [3], [4]]. Among existing modalities, ultrasound (US) imaging (USI) is widely used because it is non-ionizing, real-time, relatively inexpensive, and compatible with compact hardware [[5], [6], [7]]. USI utilizes acoustic impedance contrast between tissues but cannot reveal molecular composition or differentiate tissues of similar acoustic properties. Photoacoustic (PA) imaging (PAI) complements USI by converting optical absorption into acoustic signals, allowing hemoglobin (Hb), oxygenation, temperature, and molecular contrast to be probed at depths beyond those reachable by purely optical microscopy [[8], [9], [10], [11], [12], [13], [14], [15], [16], [17], [18], [19]]. Compared with optical imaging, where optical scattering limits the penetration depth to a few millimeters in biological tissue [[20], [21], [22]], PAI overcomes this limitation because ultrasonic scattering in biological tissue is approximately two to three orders of magnitude weaker than optical scattering. PAI can achieve centimeter-scale penetration under favorable conditions, with approximately 5–7 cm demonstrated in selected systems depending on wavelength, target contrast, illumination geometry, and detector sensitivity [23]. PAI also adds spectroscopic sensitivity to chromophores, including oxy-hemoglobin (HbO_2_) and deoxy-hemoglobin (HbR) [[24], [25], [26], [27]]. The distinct optical absorption spectra of these chromophores enable quantitative measurement of Hb concentration, blood oxygen saturation (sO_2_), temperature, and lipid content [15,24,28].

Together, USI and PAI offer a natural foundation for wearable imaging: one modality supplies anatomy and motion, and the other adds functional optical contrast. The difficulty is that conventional ultrasound systems and most laboratory PAI systems still rely on rigid probes, bulky consoles, external lasers, tethered acquisition hardware, and trained operators. These constraints limit their use for long-term, hands-free, or out-of-hospital monitoring [[29], [30], [31]]. In point-of-care and longitudinal monitoring, this combination can align anatomy and motion with vascular, oxygenation, or molecular readouts in the same examination, as demonstrated in peripheral-vascular and interventional PA/US studies [32,33] and in compact body-worn or portable PA platforms [[34], [35], [36], [37]].

Making USI and PAI wearable is, therefore, a system-level problem. Device miniaturization, flexible integration, skin-safe coupling, imaging quality, low-power operation, compact electronics, wireless transmission, and efficient reconstruction must be addressed together rather than as isolated engineering tasks. Recent work on micromachined ultrasonic transducers, soft ultrasound patches, miniaturized laser sources, integrated electronics, and computational imaging has begun to define the practical design space for wearable PA/US platforms. Representative advances include micromachined transducer miniaturization [38], bioadhesive and stretchable ultrasound patches [39,40], semiconductor PA excitation [28,41,42], integrated and wireless electronics [43,44], and spatially encoded acquisition [[45], [46], [47]].

Here, the term wearable is used in a tiered sense. Handheld, portable, or miniaturized systems that demonstrate enabling components but are neither attached to nor carried on the subject are treated as toward-wearable precursors and are not assigned a Level 1–4 classification:

• **Level 1: Wearable probe or patch.** The sensing interface adheres to the body and remains attached. Off-board instruments handle power, data acquisition, and image reconstruction. The device achieves skin conformity but not system-level autonomy. Representative Level 1 systems include bioadhesive or conformal ultrasound and PA patches that retain off-board instrumentation [39,48,49].
• **Level 2: Body-mounted module.** Some excitation, detection, or control electronics are co-located on the subject, but wired connections to external acquisition hardware remain. Examples include belt-worn amplifiers and head-mounted optical delivery units. This arrangement reduces the hardware burden on the subject but does not eliminate it. Representative Level 2 systems include head-mounted PA instruments that place the sensing head on the subject while retaining external support hardware [[50], [51], [52]].
• **Level 3: Body-worn distributed system.** The subject carries or wears the sensing head and key subsystems, such as the laser driver, data-acquisition unit, signal processor, and power supply, in modules such as a watch-type imaging head, belt unit, or backpack. The modules may remain interconnected, but the system operates while the subject moves without stationary benchtop hardware. The watch-and-backpack photoacoustic platform in [34] is a representative Level 3 system.
• **Level 4: Fully wearable platform.** A single untethered system integrates the sensing interface, light source or pulser, analog frontend, digitizer, onboard processor, battery, and wireless communication. The platform operates autonomously during natural motion with minimal operator involvement. A representative Level 4 direction is the fully integrated wireless ultrasound platform reported in [44].

This distinction matters because mechanical conformity or portability alone does not make an imaging system wearable in practice. A handheld probe or portable console remains a toward-wearable precursor unless the demonstrated configuration is attached to or carried on the subject. Many skin-conformal prototypes still depend on laboratory lasers, benchtop ultrasound consoles, cable bundles, or off-board reconstruction [34,49,53,54]. The resulting design challenge is not a simple opposition between imaging performance and wearability: a wearable patch can retain a large aperture, for example, but aperture size, channel count, interconnect density, excitation energy, acquisition bandwidth, computation, power, heat, and packaging must be balanced at the system level. The relevant question is therefore which combination of these resources is sufficient for a defined monitoring task while remaining comfortable, safe, and operationally autonomous.

**Fig. 1:**
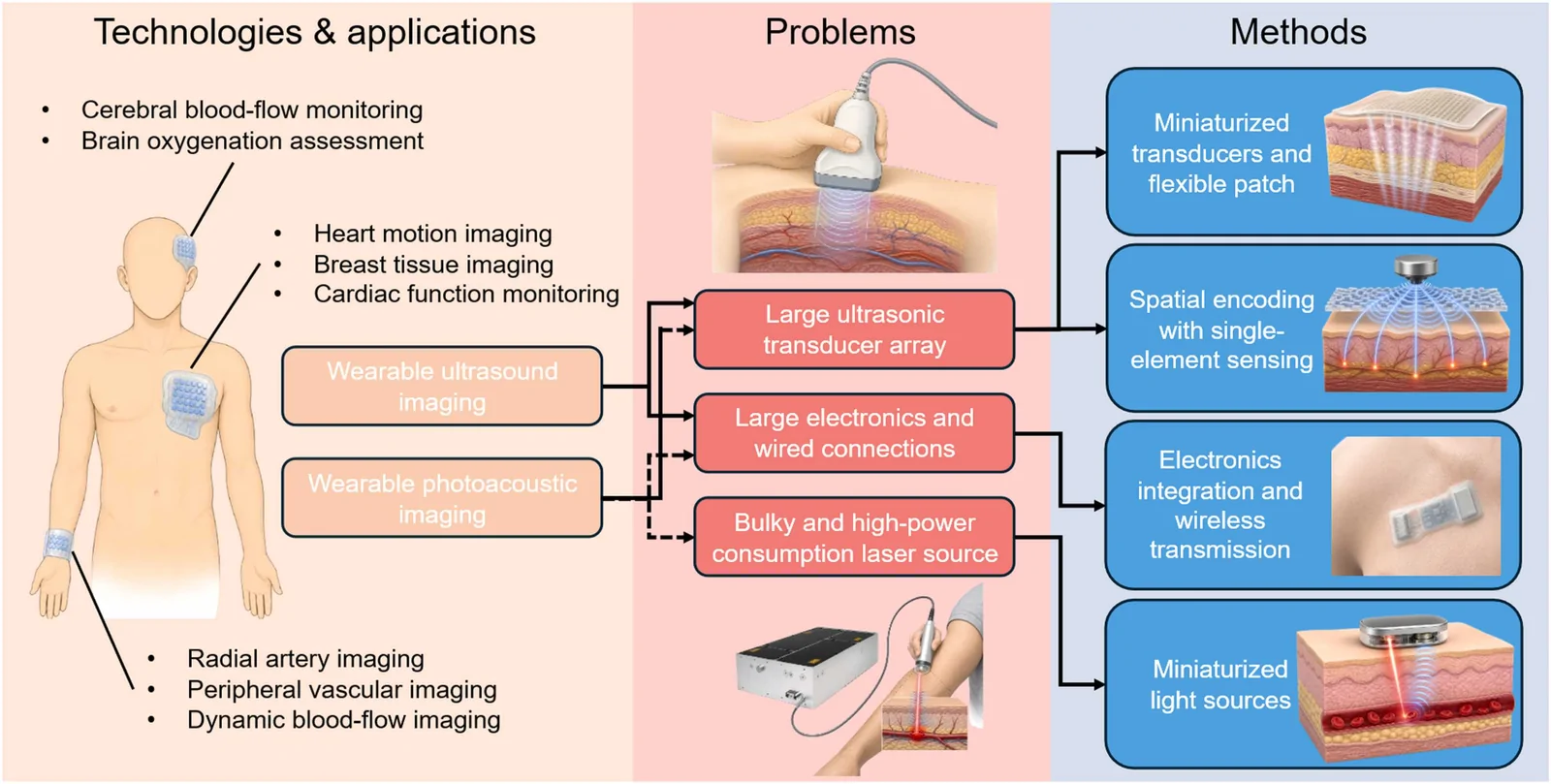
Overview of wearable ultrasound and photoacoustic imaging technologies.

This review is organized around these coupled design constraints (Fig. 1). We first trace wearable USI from conventional piezoelectric arrays and flexible or stretchable patches to miniaturized capacitive and piezoelectric micromachined transducer arrays, followed by application-specific ultrasound systems. We then review compact PA excitation sources, acoustic receivers, system architectures, and application-specific wearable or toward-wearable PAI. A separate section synthesizes multimodal PA/US integration. Finally, we examine system integration, wireless operation, spatial encoding, reconstruction, quantification, and translational requirements.

## 2 Flexible, stretchable, and micromachined transducers for wearable USI

Wearable USI has evolved from conventional piezoelectric probes toward body-conformal arrays and, more recently, micromachined transducers. The progression begins with rigid piezoelectric arrays coupled through soft adhesive interfaces, continues through flexible and stretchable piezoelectric layouts, and then extends to capacitive micromachined ultrasonic transducer (CMUT) and piezoelectric micromachined ultrasonic transducer (PMUT) technologies that offer additional opportunities for miniaturization, dense integration, and low-power electronics. This sequence separates mechanical strategies for maintaining skin contact from device-level strategies for reducing transducer and electronic footprint.

### 2.1 Conventional piezoelectric, flexible, and stretchable ultrasound patches

Ultrasound patches have become a major route toward long-duration and hands-free USI. In most designs, wearability is pursued in one of two ways: either a relatively rigid transducer array is attached to the body through a soft adhesive interface, or the transducer array and its electrodes are themselves made flexible or stretchable. The first approach tends to preserve array geometry and image quality, whereas the second improves conformability and tolerance to body motion but makes element position, crosstalk, and reconstruction more difficult to control.

**Fig. 2:**
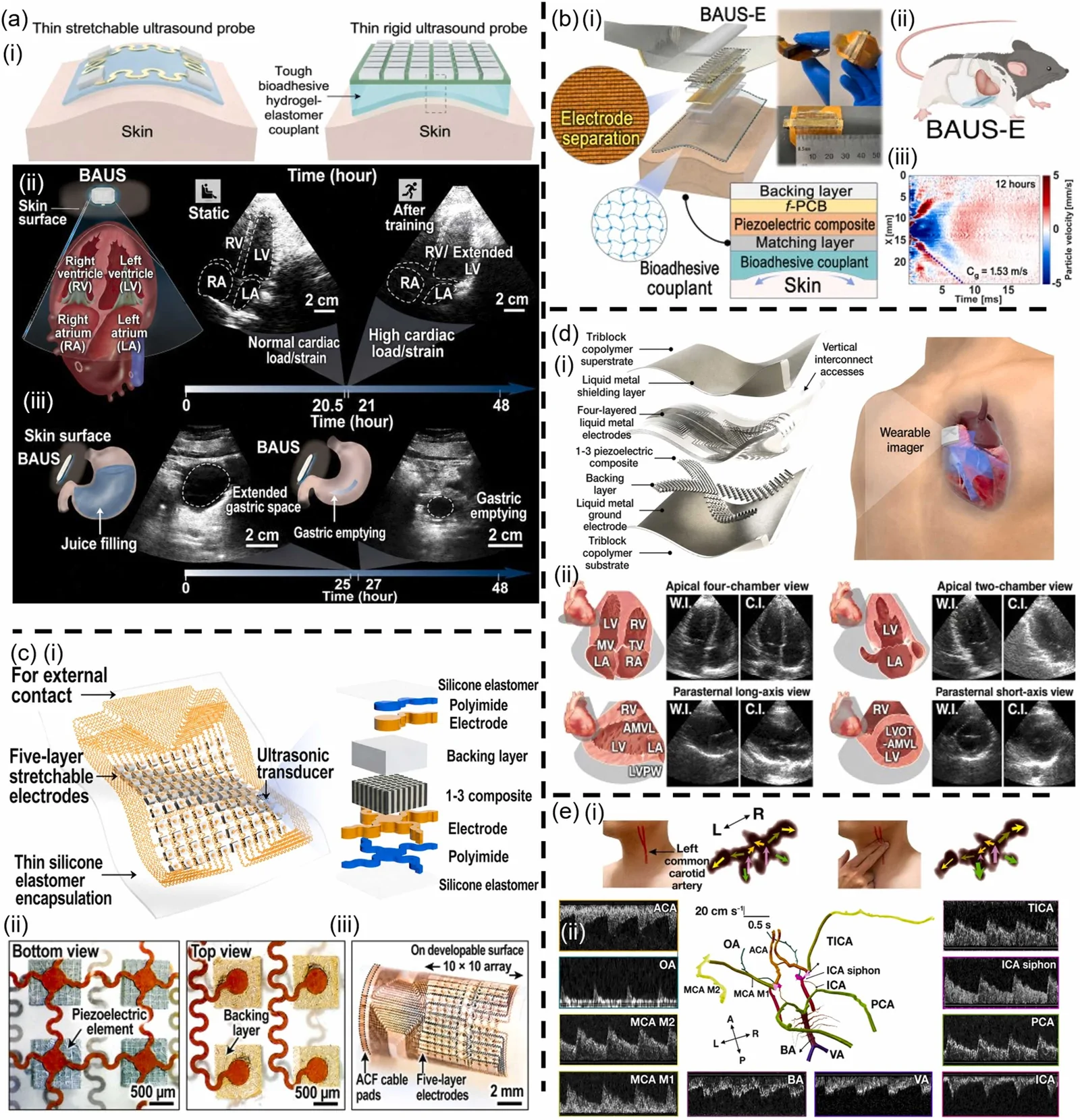
Flexible and stretchable ultrasound patches for wearable ultrasound imaging. (a) (i) Existing wearable ultrasound imaging depending on flexible and stretchable devices attached to the skin (left), and bioadhesive ultrasound device consisting of a thin and rigid ultrasound probe robustly adhered to the skin via a couplant made of a hydrogel-elastomer hybrid (right). (ii) The dynamics of the four chambers of the heart. The size of the left ventricle greatly increases after 0.5 h of physical exercise. (iii) The dynamics of the stomach. The gastric antral cross-sectional area gradually decreases after the subject drinks 450 ml of juice. BAUS: bioadhesive ultrasound. (b) (i) Schematic illustrations of the bioadhesive ultrasound patch components. (ii) The setup for evaluating elasticity changes in rats and (iii) the corresponding spatiotemporal map of shear wave velocities. BAUS-E: bioadhesive ultrasound elastography. (c) (i) Schematics showing the wearable transducer array structure. (ii) The bottom view showing piezoelectric material and bottom electrodes (left) and the top view showing backing layer and top electrodes (right). (iii) The stretchable device bent around a developable surface. (d) (i) Schematics showing the exploded view of the wearable cardiac imager. (ii) Schematics and brightness-mode (B-mode) images of cardiac anatomies from the commercial imager (C.I.) and wearable imager (W.I.). LV, left ventricle; RV, right ventricle; MV, mitral valve; TV, tricuspid valve; LA, left atrium; RA, right atrium; AMVL, anterior mitral valve leaflet; LVPW, left ventricular posterior wall; LVOT, left ventricular outflow tract. (e) (i) Volumetric power Doppler images before and during the left common carotid artery being compressed. (ii) Blood flow spectra recorded from representative arterial segments of one participant using the ultrasound patch. Reprinted with permission from [39,40,49,53,57].

Wang et al. [39] proposed a bioadhesive ultrasound probe that consists of a thin and rigid ultrasound probe robustly attached to human skin via an interface layer made of a soft and bioadhesive hydrogel-elastomer hybrid (Fig. 2**(a)-i**). This ultrasound probe had a thickness of 3 mm, length and width of 1–2 cm, and weight of 10–40 g, achieving a lateral imaging resolution of 0.38 mm at the imaging focal depth of 2 cm. It can withstand high pulling forces and maintain firm adhesion on the skin over 48 h without impairing its continuous imaging performance for various human organs (Fig. 2**(a)-ii,iii**). Liu et al. [53] further extended the bioadhesive strategy to wearable shear wave elastography. The bioadhesive ultrasound elastography device was integrated with a bioadhesive couplant for stable attachment to the skin (Fig. 2**(b)-i**). This device enabled hands-free elastographic measurements in small-animal models (Fig. 2**(b)-ii**), while the corresponding particle velocity maps demonstrated its capability to estimate tissue stiffness during long-term monitoring (Fig. 2**(b)-iii**). Other high-performance adhesive interfaces for wearable ultrasound devices have also been extensively studied [[54], [55], [56]], aiming to develop soft, tough, and anti-dehydrating materials suitable for complex surfaces and capable of long-term adhesion. Comparatively, flexible ultrasound patches discussed below are troubled by low imaging quality. Since high-quality imaging relies on accurate knowledge of transducer element positions in the ultrasonic transducer array, stretching such an array will significantly damage its imaging performance.

So far, most ultrasound patches are wholly flexible, stretchable, and conformal, conforming to curved surfaces of human skin without needing soft interfaces and showing better adaptability to human movement. In 2018, Hu et al. [40] designed and fabricated a stretchable ultrasonic patch with an array of 10 × 10 ultrasonic transducers (Fig. 2**(c)-i**). The transducer array was connected by an island-bridge structured matrix (Fig. 2**(c)-ii**), so that the matrix is rigid individually but soft globally (Fig. 2**(c)-iii**), offering more than 50% biaxial stretchability without impairing imaging results. This novel structure successfully broke the limitations of previous exploration regarding flexible ultrasonic devices, including using organic piezoelectric films [[58], [59], [60]], which have good flexibility but low electromechanical coupling coefficients and low Curie points, and embedding piezoelectric ceramic into polymer substrates [61,62], which cannot conform to curved surfaces without external forces applied. Three years later, they further improved the performance of such ultrasound patches [48] by decreasing the resonance frequency of ultrasonic transducers from 7.5 to 2 MHz and reducing the element pitch of the transducer array from 2.5 mm × 2 mm to 0.8 mm × 0.8 mm, thus achieving a higher acoustic energy density for deep tissue penetration. This elastic device was attached to the human body to detect ultrasound Doppler signals from human left and right ventricles as well as carotid arteries and jugular veins *in vivo*. However, the *λ*/2-pitch principle [63,64] was not satisfied within such transducer arrays, as dicing of the piezoelectric composite materials to millimeter-sized dimensions is very challenging with existing facilities. In contrast, such dimensions are easier to achieve by using the micromachining fabrication process, making PMUTs powerful alternatives to traditional ultrasonic transducers in ultrasound patches.

### 2.2 Micromachined PMUT and CMUT arrays

With the development of microelectromechanical systems (MEMS) technology, micromachining has enabled the miniaturization of ultrasonic transducers. These micromachined ultrasonic transducers (MUTs) can be divided into two categories: CMUTs and PMUTs. MUTs have the advantages of lower cost, lower power consumption, smaller size, more design flexibility, and better acoustic impedance coupling, and they are well suited for array integration and batch fabrication [38]. With all these promising features, MUTs have been considered as suitable candidates for wearable USI devices.

**Fig. 3:**
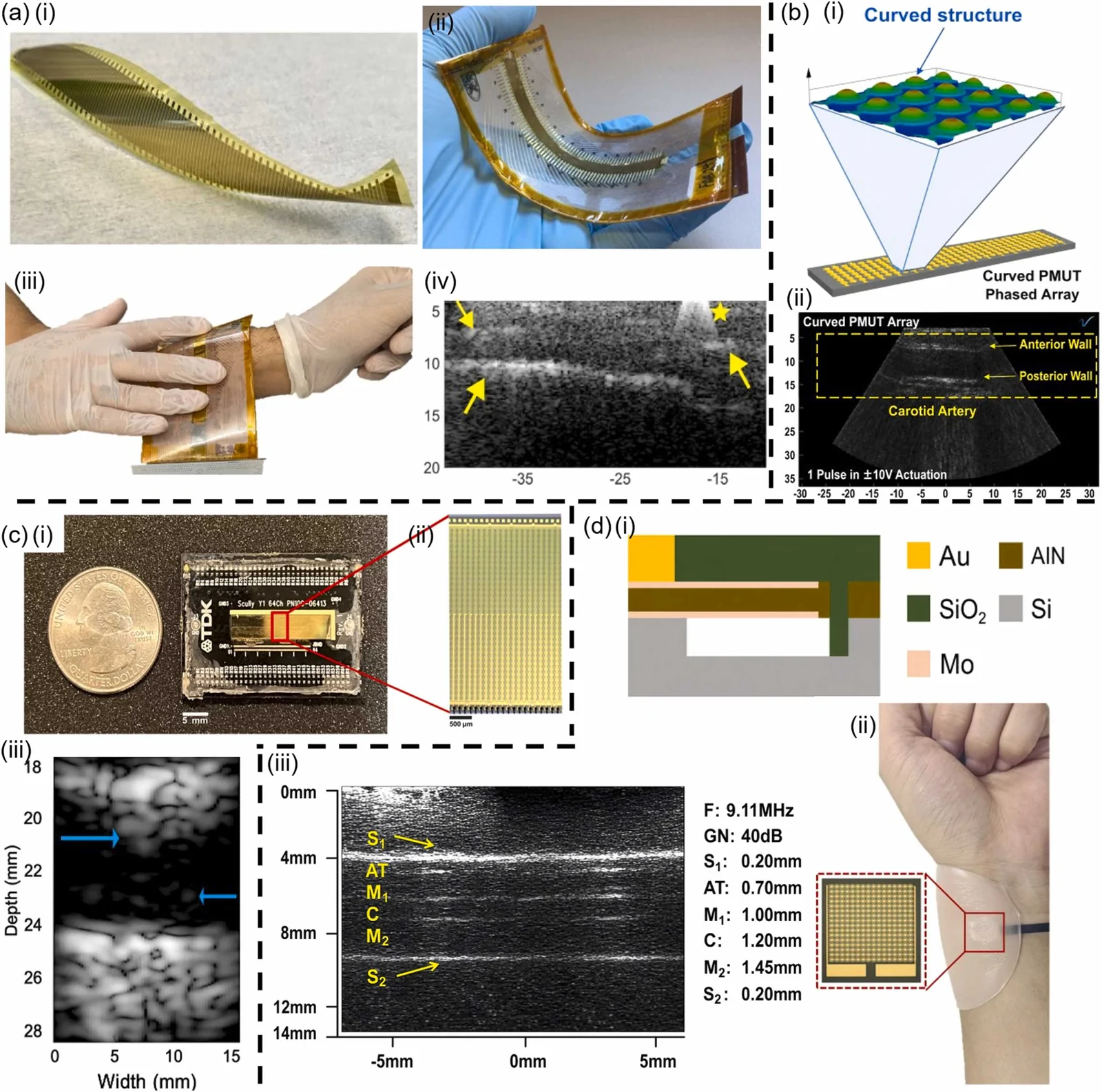
Wearable CMUT and PMUT arrays for conformal ultrasound imaging and monitoring. (a) (i) A long monolithic flexible CMUT array. (ii) CMUT array packaged assembly with a polydimethylsiloxane (PDMS) coating for *in vivo* experiments. (iii) B-scan of a forearm acquired with the array in an unknown shape and (iv) reconstructed image assuming a flat geometry. Arrows indicate detected tissue structures. Units: mm. (b) (i) Low-voltage driven wearable ultrasound phased array and (ii) the corresponding carotid artery image. Unit: mm. (c) (i) Imaging face of the miniaturized PMUT array (27 mm × 36 mm) and (ii) magnified view of the individual PMUTs shorted together in columns. (iii) B-mode image of the common carotid artery captured with the PMUT array. (d) (i) Cross-sectional view of the high-frequency and wideband PMUT cell. (ii) PMUT array fixed on a wrist with a commercial silicone patch. (iii) B-mode image of a porcine ear captured by the PMUT array. Reprinted with permission from [65,[67], [68], [69]].

Flexible CMUT arrays have been preliminarily investigated for conformal and large-area wearable USI. A long monolithic flexible CMUT array was proposed for pulse-echo imaging [65], where the array was fabricated on a bendable substrate and could maintain its structure under different curvatures (Fig. 3**(a)-i,ii**). This device was further attached to curved surfaces and used for pulse-echo imaging of superficial tissue structures (Fig. 3**(a)-iii,iv**), demonstrating the feasibility of flexible CMUTs for body-conformal and hands-free USI. In addition, a flexible CMUT-based ultrasound transducer array with statically adjustable curvature was reported for anti-inflammatory treatment [66]. Although this work mainly focused on therapeutic ultrasound, the curvature-adjustable structure provides an effective strategy to improve acoustic coupling and beam control on curved biological surfaces, which is also beneficial for future wearable USI systems.

Compared with PMUTs, the aforementioned CMUTs feature higher electromechanical coupling coefficients and larger bandwidths [38,70], but many CMUT architectures require a direct-current (DC) bias and careful electrical isolation. PMUTs can operate at lower voltages and offer fewer geometric constraints, making them attractive for integration with low-voltage electronics and complementary metal–oxide–semiconductor (CMOS)-compatible systems [63,71,72]. Therefore, PMUT arrays have recently attracted increasing attention as promising candidates for wearable USI.

Taken together, the MUT literature shows a progression from device-level improvements in bandwidth, electromechanical coupling, and array fabrication [38,63,[70], [71], [72]], to flexible or curvature-adaptable CMUT arrays [65,66], and finally to task-oriented PMUT prototypes for carotid and superficial-tissue imaging [[67], [68], [69]]. The transition toward wearability therefore depends not on miniaturization alone, but on combining low-voltage operation, dense arrays, conformal packaging, compact electronics, and *in vivo* validation.

Several PMUT-based arrays have been demonstrated for wearable vascular and superficial tissue imaging. Ma et al. [67] proposed a low-voltage driven wearable ultrasound phased array based on a highly sensitive curved PMUT array (Fig. 3**(b)-i**). Benefiting from the curved membrane structure, this array enabled carotid artery imaging under low-voltage excitation (Fig. 3**(b)-ii**), showing its potential for wearable cardiovascular monitoring. A miniaturized PMUT array was further investigated for *in vivo* characterization of central arterial properties [68] and compared with a clinical transducer (Fig. 3**(c)-i,ii**). Although the image quality was still lower than that of the commercial probe, carotid pulse wave imaging and arterial property estimation were successfully demonstrated (Fig. 3**(c)-iii**), indicating the feasibility of compact PMUT arrays for continuous vascular assessment. For superficial tissue applications, Wei et al. [69] developed high-frequency and wideband PMUTs for skincare assessment (Fig. 3**(d)-i,ii**). The flexible PMUT patch was able to resolve layered skin-like structures and *ex vivo* tissues (Fig. 3**(d)-iii**), suggesting its potential for wearable dermatological imaging and cosmetic evaluation.

### 2.3 Wearable ultrasound imaging applications

With continued improvements in ultrasound patches, wearable USI has been applied to cardiovascular, cerebral, bladder, breast, and other targets. For cardiovascular imaging, Hu et al. [57] reported a wearable cardiac ultrasound device for continuous, real-time monitoring (Fig. 2**(d)-i**). The device used liquid-metal high-density multilayer electrodes and a thermoplastic elastomer substrate, providing stretchability, skin conformity, high electromechanical coupling, and low crosstalk. It enabled hands-free views of cardiac anatomy (Fig. 2**(d)-ii**). Chen et al. [73] further improved stretchability using silver nanowires with a composite elastic substrate and reported axial and lateral resolutions of approximately 0.31 and 0.34 mm at a 1-cm imaging depth. Because silver nanowires can be screen-printed into dense, stable stretchable electrodes, they are a practical candidate for future wearable ultrasound arrays [74,75].

For monitoring cerebral hemodynamics and brain activities, a wearable transcranial Doppler (TCD) system was proposed by Pietrangelo et al. [76], but *in vivo* experiments are still necessary for comparison with commercial TCD systems. Zhou et al. [49] presented hands-free volumetric imaging and continuous monitoring of cerebral blood flow based on a conformal ultrasound patch for the first time. The device is 20 × 28 × 1.3 mm^3^ in size and 0.945 g in weight, featuring a measurement success rate close to that of commercial TCD probes. With diverging waves to image the entire arterial network (Fig. 2**(e)-i**) and focused waves to monitor blood flow spectra at target arterial areas (Fig. 2**(e)-ii**), ultrafast imaging algorithms greatly enhanced the imaging signal-to-noise ratio (SNR), though the spatial resolution is limited.

For other tissue imaging applications, Zhang et al. [77] reported a conformable phased-array ultrasound patch for bladder volume monitoring, which consisted of five subarrays and provided B-mode imaging and measurement without the need for transducer motion. Pu et al. [78] further simplified bladder volume monitoring systems by designing and fabricating a wearable ultrasound patch based on a 4 × 4 lead zirconate titanate (PZT) transducer array. This patch was only tested using balloon-bladder phantoms and clinical trials are still required to determine the accuracy of bladder volume measurement *in vivo*. Du et al. [79] utilized an ultrasound patch for breast tissue imaging for the first time, where a Yb/Bi-doped lead indium niobate–lead magnesium niobate–lead titanate (PIN-PMN-PT) single crystal with superior properties was studied and exploited as an alternative to traditional PZT ceramics. With a scanning trace physically guiding 360° rotation of each transducer, the one-dimensional (1D) array can fully cover the entire breast surface and obtain comprehensive reconstructed images.

Table 1 compares representative ultrasound systems, with one study per row using the same system-level metrics and assigns wearability from the demonstrated configuration rather than the intended future use. Most body-adhered probes remain Level 1 because acquisition and processing are off-board, whereas the integrated wireless platform in [44] reaches Level 4. NR denotes information not reported in the cited study.

**Table 1:** Study-level comparison of representative wearable and toward-wearable ultrasound imaging systems.

**Study**	**Status**	**Transducer / channels**	**Central frequency**	**Size / weight**	**Connection / battery life**	**Frame rate**	**Resolution / SNR**	**Imaging depth**	**Validation**
[39]	Level 1	Rigid piezoelectric array on soft substrate / 80	10 MHz	1–2 cm lateral; 3 mm thick / 10–40 g	Wired / NR	40 Hz	~0.38 mm / NR	> 60 mm	Long-term organ imaging (n = 15)
[40]	Level 1	Flexible piezoelectric array / 10 × 10	7.5 MHz	NR / NR	Wired / NR	NR	~0.61 mm / ~20.28 dB	Up to 60 mm	Conformal structural imaging (n = NR)
[48]	Level 1	Flexible piezoelectric array / 12 × 12	2 MHz	0.65 mm thick	Wired / NR	NR	~2.5 mm / > 18 dB	Up to 140 mm	Cardiac and vascular Doppler (n = 20)
[49]	Level 1	Flexible piezoelectric array / 16 × 16	2 MHz	20 × 28 × 1.3 mm³ / 0.945 g	Wired / NR	200 Hz	NR / NR	40–100 mm	Cerebral hemodynamics (n = 36)
[65]	Level 1	Flexible CMUT array / 128	5.84 MHz	91 mm × 10.7 mm	Wired / NR	NR	~1.03 mm / NR	Up to 15 mm	Finger and forearm imaging (n = 2)
[80]	Level 1	Flexible CMUT array / 64	4.25 MHz	NR / NR	Wired / NR	NR	~0.55 mm	~70 mm	Carotid artery imaging (n = 9)
[68]	Handheld	Miniaturized PMUT array / 64	4.8 MHz	36 mm × 27 mm	Wired / NR	2790 Hz	NR / NR	NR	Carotid artery imaging (n = 9)
[81]	Level 1	Miniaturized PMUT array / 64	7 MHz	10 mm × 1.5 mm	Wired / NR	NR	~0.26 mm / 28.2 dB	> 30 mm	Carotid artery, thyroid gland, and dorsalis pedis artery imaging (n = 5)
[44]	Level 4	Flexible piezoelectric array / 32	4 MHz	96.6 × 43.5 × 6.5 mm³ / NR	Wireless / 12 h	Up to 1 kHz	~0.33 mm / NR	Up to 164 mm	Deep-tissue motion monitoring (n = 10)

## 3 Wearable and toward-wearable photoacoustic imaging systems

Wearable PAI faces a stricter integration problem than wearable USI because optical excitation must be brought to the body together with acoustic detection and coupling. The main hardware directions are compact semiconductor light sources, flexible or miniaturized ultrasonic receivers, and packages that keep the optical and acoustic paths aligned during motion and repeated use.

### 3.1 Miniaturized optical excitation sources

Laser diodes (LDs) are attractive for miniaturized PAI because they are compact, efficient, and capable of high repetition rates and multi-wavelength integration [82,83]. Compared with Q-switched optical parametric oscillator (OPO) lasers, however, LDs generally provide lower pulse energy and more limited penetration depth. Wearable PAI systems based on LDs therefore depend heavily on optical design, diode-array scaling, detector sensitivity, and reconstruction methods that can work with lower signal levels.

Early LD-based PAI systems were usually portable or handheld rather than wearable, but they established several useful building blocks. Zeng et al. first reported a cost-effective LD-based three-dimensional (3D) PAI system [84] and later developed an LD-based photoacoustic microscopy (PAM) system with improved lateral resolution [41]. A compact handheld PA probe was also demonstrated by integrating an ultrasound transducer array with an 805 nm LD source [85]. Erfanzadeh et al. [86] presented a 905 nm pulsed-LD-based photoacoustic tomography (PAT) system with optimized optical collimation and focusing, followed by a galvanometer-scanned LD-based PAM system for *ex vivo* mouse-ear vasculature. Visible LD-based and continuous-wave LD (CWLD)-based systems were later reported by Deng et al. [87] and Li et al. [88], enabling *in vivo* mouse-ear microvascular imaging with simplified excitation configurations. These systems did not yet solve the wearable system problem, but they showed that PA imaging could move away from large benchtop lasers.

**Fig. 4:**
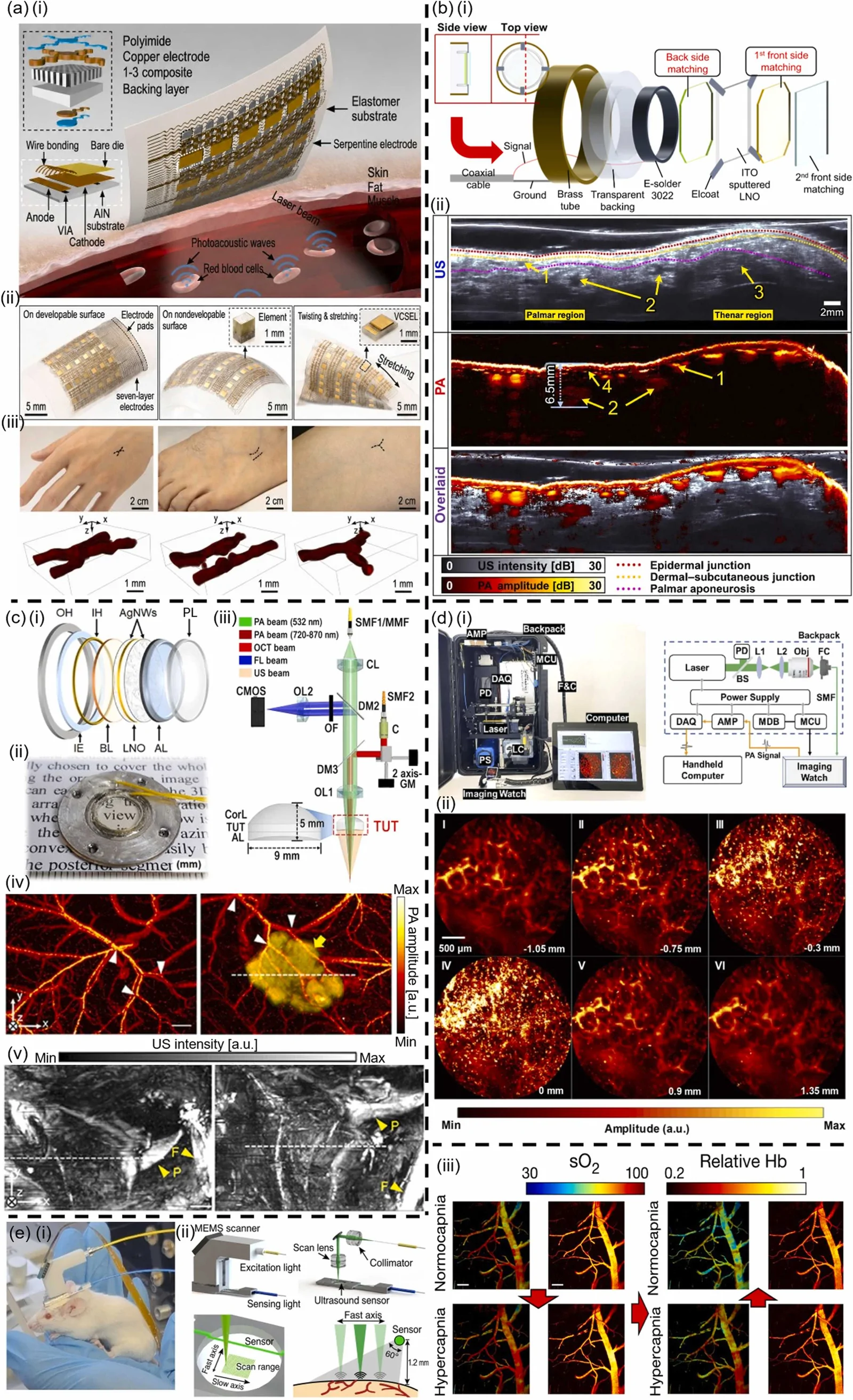
Wearable photoacoustic imaging devices. (a) (i) Schematics of the soft photoacoustic patch structure and the working principle. (ii) Photos of the patch under different modes of deformation. (iii) Photos and corresponding reconstructed 3D photoacoustic images of the hand, foot, thigh, and forearm. (b) (i) Structural schematic of the transparent transducer. (ii) Two-dimensional (2D) cross-sectional PA/US images of a human palm. 1, palmar vein; 2, common palmar digital artery; 3, adductor pollicis; 4, subcutaneous vessels. (c) (i) Schematic illustration of the layer-by-layer transparent transducer. OH, outer housing; IE, insulation epoxy; IH, inner housing; BL, backing layer; AgNWs: silver nanowires; AL, acoustic lens; PL, parylene coating layer. (ii) Photograph demonstrating the transparency with an element size of 9 mm. (iii) Schematic of an integrated quadruple fusion imaging head module using a transparent transducer: PAI, USI, OCT, and FLI. SMF, single-mode fiber; MMF, multimode fiber; CL, collimation lens; OL, objective lens; OF, optical filter; DM, dichroic mirror; C, collimator; GM, galvanometer; CorL, correction lens; TUT, transparent ultrasonic transducer; AL, acoustic lens. (iv-v) *In vivo* multimodal imaging of subcutaneous melanomas in a mouse model. (iv) PA maximum-amplitude-projection (MAP) images before (left) and on day 3 after (right) melanoma injection. (v) US maximum intensity projection (MIP) images before (left) and after (right) melanoma injection. Scale bar: 1 mm. (d) (i) Photo and schematics of the wearable photoacoustic watch system. AMP: amplifier; MCU: microcontroller unit; DAQ: data acquisition card; F&C: fiber and cable; PD: photodiode; PS: power supply; LC: laser controller; BS: beam splitter; L: lens; Obj: objective lens; FC: fiber coupler; SMF: single-mode fiber; MDB: motor drive board. (ii) Photoacoustic images of the human wrist with a shifted laser focus. (e) (i) A freely moving mouse wearing the photoacoustic imaging probe. (ii) External (top left) and internal (top right) structure of the imaging probe. Stereoscopic (bottom left) and side (bottom right) views of the laser beam scanning and ultrasound detection schemes. (iii) Hb and sO_2_ photoacoustic images in freely moving mice in a hypercapnia normocapnia cycle. Scale bar: 200 μm. Reprinted with permission from [28,34,51,91,92].

Among LD technologies, vertical-cavity surface-emitting lasers (VCSELs) are especially relevant for wearable PAI because they emit perpendicular to the chip surface and can be fabricated as two-dimensional arrays [89,90]. This geometry supports compact packaging, broader illumination, and integration with soft electronic layouts. Gao et al. [28] reported a soft photoacoustic patch that combined high-power VCSEL arrays and piezoelectric ultrasonic transducers on a common soft substrate (Fig. 4**(a)-i, ii**). The patch mapped Hb at centimeter-scale depths and used the linear relationship between PA amplitude and temperature for core-temperature monitoring. Its main contribution was the direct integration of optical excitation and acoustic detection in a skin-conformal PA platform.

Other VCSEL-based systems have explored smaller sensing modules and cardiovascular readouts. Panchawagh et al. [93] proposed a compact PAI module for wearable health monitoring devices, using a VCSEL excitation unit and an ultrasonic detection module sized for future wrist-worn integration. Fang et al. [94] proposed a wearable PA sensing system for noninvasive blood-pressure monitoring. In that design, the VCSEL pulse width, transducer length, and frequency bandwidth were optimized to detect vessel-related PA signals in vascular models. These studies are still early, particularly with respect to *in vivo* validation and autonomous operation, but they show why semiconductor light sources are central to wearable PAI.

In contrast to LDs and VCSELs, light-emitting diodes (LEDs) are non-laser semiconductor emitters based on spontaneous emission. LEDs provide another important route toward miniaturized and wearable PAI. Compared with LDs, LEDs generally have lower per-pulse energy, broader spectral bandwidth, and lower optical fluence, which limits penetration depth and SNR. However, they offer several practical advantages that are highly attractive for wearable and commercial systems, including low cost, compact size, low power consumption, simple driving electronics, reduced laser-safety requirements, and straightforward integration with conventional ultrasound probes [95,96]. Their high pulse repetition rates also allow extensive signal averaging to compensate for the lower pulse energy and improve PA signal SNR, making LED-based PAI particularly suitable for superficial vascular, lymphatic, and hemodynamic monitoring applications [32,97].

Early LED-based studies established the feasibility of replacing bulky pulsed lasers with compact semiconductor illumination. Dai et al. demonstrated *in vivo* vascular imaging using a low-cost miniature LED excitation source, showing that LED-based PAI could visualize mouse-ear vasculature without a conventional laser [98]. Hariri et al. characterized a portable LED-based PAI system in which two LED arrays were mounted on the sides of a conventional ultrasound transducer, and demonstrated phantom imaging, exogenous-contrast detection, and *in vivo* molecular imaging in small animals [95]. Xia et al. further developed a handheld real-time LED-based PA/US system for guiding minimally invasive procedures, achieving visualization of superficial human finger and wrist vasculature and improved needle contrast relative to ultrasound alone [33]. Zhu et al. reported LED-based PA/US imaging for human peripheral microvasculature, arterial pulsation, blood reperfusion, and oxygen saturation, highlighting its potential for point-of-care vascular assessment [32].

More recent studies have moved LED-based PAI closer to wearable and clinical use cases. Bulsink et al. implemented dual-wavelength LED-based PA oxygen-saturation imaging with fluence compensation and demonstrated imaging in phantoms, small animals, and a human subject at superficial depths [99]. Van Heumen et al. applied LED-based PAI for preoperative visualization of lymphatic vessels in patients with secondary limb lymphedema, showing that lymphatic vessels and veins could be differentiated even in the presence of dermal backflow [100]. Although fully wearable LED-based PAI is still less mature than handheld LED-PA/US systems, recent wearable LED-array concepts using frequency-modulated excitation and microlens-based light concentration suggest a promising path toward skin-conformal, low-cost PA monitoring platforms [42]. Overall, LEDs complement LDs and VCSELs by prioritizing affordability, safety, and manufacturability, but future wearable systems will require improved light delivery, detector sensitivity, coded excitation, and reconstruction methods to overcome their lower pulse energy.

### 3.2 Compact acoustic receivers and integrated PA sensing architectures

The acoustic receiver is equally important. It determines sensitivity, bandwidth, imaging depth, spatial resolution, and the degree to which the PA sensing head can be miniaturized. For wearable PAI, detector design also affects mechanical stability, coupling, packaging thickness, and the ability to share hardware with ultrasound imaging.

From this perspective, the photoacoustic patch reported by Gao et al. [28] is useful because it integrates light delivery, acoustic detection, and soft mechanics in one platform (Fig. 4**(a)-i,ii**). The reconstructed vascular images of the hand, foot, thigh, and forearm demonstrate body-conformal 3D PAI across several anatomical sites (Fig. 4**(a)-iii**).

PMUTs are also being explored as compact PA receivers. Dangi et al. [101] fabricated a linear PZT-based PMUT array and integrated it with a customized circuit board and optical fiber bundle for phantom PAI. Although the system was not wearable, it demonstrated that PMUT arrays can provide broad PA bandwidth in a compact detector format. Fang et al. [102] later combined a high-fill-factor PMUT array with a compact VCSEL source to form a miniature PA sensing system. PMUT-VCSEL integration is appealing because both the excitation and detection modules can, in principle, be packaged into a small wearable footprint.

Transparent ultrasonic transducers provide another promising strategy for compact and wearable PAI because they allow optical excitation to pass through the acoustic receiver. In conventional PA probes, opaque transducers usually require side illumination or off-axis optical delivery, which increases probe size and can introduce optical-acoustic misalignment. Transparent transducers enable a more coaxial configuration, improving the overlap between optical illumination and acoustic detection while simplifying the probe geometry [91,[103], [104], [105], [106]]. Transparent transducers can be constructed with optically transparent acoustic stacks (Fig. 4**(b)-i**), allowing light to pass through the receiver while maintaining acoustic detection [91]. This configuration has enabled co-registered US and PA imaging of human palm structures, where anatomical layers and vascular features can be visualized in the same cross-sectional view (Fig. 4**(b)-ii**). Beyond single-modality PAI, transparent transducers have also been used to integrate ultrasound, photoacoustic, optical coherence tomography (OCT), and fluorescence imaging (FLI) in a single optical-acoustic platform [92]. Through a transparent acoustic aperture (Fig. 4**(c)-i,ii**), the corresponding quadruple fusion imaging head integrates PAI, USI, OCT, and FLI (Fig. 4**(c)-iii**). *In vivo* multimodal imaging of subcutaneous melanoma further demonstrates the ability of this design to combine vascular PA contrast with structural US information (Fig. 4**(c)-iv,v**). Transparent LiNbO_3_ linear arrays and transparent CMUT arrays further extend this concept to array-level, real-time multimodal PA/US imaging [107,108].

For wearable PAI, transparent transducers are particularly attractive because they can support backside illumination from LED, LD, or VCSEL sources while maintaining a thin and compact skin-contact imaging head. Such a configuration could reduce the lateral footprint of the optical delivery module, improve optical-acoustic alignment during body motion, and facilitate co-registered monitoring of superficial vasculature, oxygenation, wounds, and peripheral circulation. In addition, optical ultrasound sensing studies using transparent transducers in wearable and mobile device formats suggest that transparent acoustic apertures may help combine optical and acoustic measurements within small form factors [109]. However, practical wearable implementation remains challenging. Optical transparency must be balanced with acoustic sensitivity, bandwidth, electrode conductivity, acoustic matching, backing-layer design, and mechanical compliance. Most existing transparent transducers are still rigid and have been demonstrated mainly in microscopy, phantom, or animal studies.

Taken together, the source and receiver technologies define a coupled trade space rather than independent design choices. LDs and VCSELs generally provide higher optical fluence and greater penetration than LEDs, but require more demanding drivers, thermal management, and laser-safety control [41,[84], [85], [86], [87], [88]]. LEDs favor low cost, compact packaging, high repetition rates, and simpler source integration, but their lower per-pulse energy usually confines imaging to superficial targets or requires extensive averaging [33,[98], [99], [100]]. Flexible and stretchable piezoelectric arrays improve skin conformity and coupling during motion, although deformation can alter element geometry and complicate interconnect design and beamforming [39,40,57]. Conventional piezoelectric arrays provide high acoustic sensitivity and established beamforming, whereas PMUTs offer lower-voltage operation and easier miniaturization but still face sensitivity and bandwidth trade-offs [28,67,101,102]. Transparent transducers simplify coaxial optical-acoustic integration and reduce probe footprint, although transparency must be balanced against acoustic sensitivity, bandwidth, and mechanical compliance [91,103,104,[106], [107], [108]]. Consequently, superficial oxygenation monitoring may favor dual-wavelength LED or VCSEL modules, whereas deeper PA imaging generally requires higher-fluence excitation and more sensitive acoustic receivers.

### 3.3 Application-specific wearable and toward-wearable PAI implementations

Wearable and toward-wearable PA systems target different applications and should not be evaluated as a single device class. Macroscopic and array-based systems prioritize field of view (FOV), penetration depth, and repeated hemodynamic measurements, whereas microscopy-based systems prioritize microvascular resolution and metabolic contrast, often with shallower penetration, smaller fields of view, and an optical or mechanical scanning burden. Distinguishing these regimes makes comparisons of resolution, depth, acquisition speed, and wearability more meaningful.

Macroscopic implementations are being developed for human vascular, musculoskeletal, and preclinical neuroimaging applications. Zhang et al. [34] proposed a photoacoustic watch for human hemodynamic monitoring, consisting of a watch-type imaging head, a handheld computer, and a backpack module containing the laser, power supply, and control electronics (Fig. 4**(d)-i**). Although the backpack prevents fully untethered operation, the watch head provides a compact wrist interface for imaging microvasculature at different depths (Fig. 4**(d)-ii**). Bing et al. [110] developed a curved-array wearable PA/US system whose geometry is suited to limb imaging and may support perfusion and structural monitoring in sports medicine or rehabilitation. For non-microscopic preclinical imaging, Tang et al. [50] used a curved transducer array, liquid light-guide interface, and head mount to perform three-dimensional PAT in behaving rats. Across these systems, the primary design priorities are stable optical-acoustic alignment, broad anatomical coverage, and reduced dependence on stationary instrumentation.

Microscopy-based implementations pursue microvascular and metabolic measurements at higher spatial resolution. Compact LD-based PAM [41,86], transparent-transducer optical-resolution PAM [106], and portable PAM architectures [36] established relevant excitation, detection, and scanning components. Head-mounted systems have extended this direction to freely moving animals: Zhong et al. [51] reported a photoacoustic fiberscope for cerebral oxygenation and hemodynamic imaging (Fig. 4**(e)-i,ii**), including Hb and sO_2_ maps under different physiological states (Fig. 4**(e)-iii**), while Liang et al. [52] developed ultrafast wearable PA microscopy for monitoring cerebral oxygen metabolism during behavior. Because these microscopy systems generally retain a limited field of view and scanning or calibration requirements, their wearability and use cases should be assessed separately from array-based PA/US and tomographic systems.

### 3.4 Multispectral and multi-wavelength wearable PAI

Quantitative oxygen-saturation imaging requires measurements at two or more wavelengths to separate oxyhemoglobin and deoxyhemoglobin contributions. Dual-wavelength LED-based PAI has demonstrated fluence-compensated sO_2_ imaging in phantoms, small animals, and a human subject [99], while head-mounted multi-wavelength PA microscopy has enabled cerebral Hb and sO_2_ mapping in freely moving mice [51]. These studies establish the feasibility of multispectral measurements in compact platforms, but they also expose integration requirements that are less important for single-wavelength structural imaging.

For a wearable system, multiple emitters must be co-packaged or wavelength-multiplexed, and their pulse energies, illumination profiles, and timing must be monitored separately. Because tissue attenuation and permissible exposure vary with wavelength, raw PA amplitudes cannot be compared directly without wavelength-dependent fluence correction. Sequential wavelength switching also creates motion artifacts when the patch shifts or the tissue moves between frames. Practical designs should therefore use rapid interleaved excitation, synchronized acquisition, per-wavelength pulse-energy normalization, image registration, and model-based or learned fluence compensation. These requirements increase source, driver, calibration, and processing complexity and represent a central barrier to reliable wearable sO_2_ imaging.

## 4 Multimodal wearable PA/US integration

The compact LD, VCSEL, and LED sources discussed in Section 3.1 and the acoustic receiver and system architectures discussed in Section 3.2 form the two hardware pillars of multimodal wearable PA/US integration. USI provides tissue boundaries, motion, and Doppler flow information, whereas PAI reports optical absorption associated with Hb concentration, oxygen saturation, temperature, contrast agents, lipids, or molecular probes. Their combination can place functional PA signals within an anatomical US frame for longitudinal monitoring. This section therefore synthesizes how the preceding source and detector technologies can share an aperture, couplant, package, synchronization scheme, and reconstruction workflow, rather than treating dual-modality imaging as the simple juxtaposition of two instruments. The multispectral considerations discussed in Section 3.4 further determine whether oxygenation can be quantified reliably during body motion.

For wearable devices, multimodal integration is not simply a matter of placing two instruments side by side. The acoustic aperture, optical delivery path, skin couplant, mechanical package, safety limits, and reconstruction workflow have to be designed as one system. A shared transducer array can reduce size, for example, but optical access must be preserved without compromising acoustic coupling. Increasing optical pulse energy can improve PA signal strength, but it also raises demands on thermal management, skin and eye safety, and battery capacity.

Existing systems illustrate distinct stages of integration. Soft PA patches [28] and the curved body-conformal PA/US configuration in [110] are Level 1 because the sensing interface is worn while substantial acquisition hardware remains off-board. The watch-and-backpack system in [34] is Level 3 because its key modules are carried by the subject. By contrast, the handheld LD/US probe in [85] and compact module intended for future wrist integration [93] are toward-wearable precursors rather than Level 1–4 systems. Head-mounted small-animal PA systems [[50], [51], [52]] are Level 2. No reviewed dual-modal human PA/US platform yet demonstrates Level 4 autonomy, mainly because compact light delivery, PA calibration, and synchronized multimodal acquisition remain difficult to combine in a soft, low-power package.

A practical path forward is to design PA/US systems around specific monitoring tasks rather than pursue a universal wearable imager. Cardiovascular applications may prioritize vessel diameter, pulse-wave dynamics, blood oxygenation, and cuff-free pressure estimation. Neurovascular applications may emphasize flow, oxygenation, and motion-robust coupling. Sports medicine and rehabilitation may combine US structural imaging with PA assessment of perfusion or inflammation. These use cases lead to different choices of wavelength, transducer frequency, aperture, frame rate, reconstruction method, and form factor.

Table 2 uses the same one-system-per-row structure for PA and PA/US platforms. It distinguishes body-attached or body-carried systems from handheld and portable toward-wearable precursors and reports the same hardware, performance, power, connection, and validation fields for every study. The comparison shows that no reviewed human PA/US platform yet reaches Level 4; NR denotes information not reported in the cited study.

**Table 2:** Study-level comparison of representative wearable and toward-wearable PA and PA/US systems.

**Study**	**Status**	**Receiver frequency / channels**	**Optical source**	**Size / weight**	**Connection / battery life**	**Resolution / SNR**	**Imaging depth**	**Validation**
[28]	Level 1	2.4 MHz / 15 × 16	VCSELs; 850 nm; 200 ns; 3 kHz	20 × 16 × 1.2 mm^3^ / NR	Wired to Verasonics Vantage 256 / NR	~0.7 mm / ~26.8 dB	> 11 mm	Blood vessels imaging (n = 1)
[37]	Level 1	2.25 MHz / 72	LDs; 450, 638, and 808 nm	80 × 25 × 24 mm^3^ / 21 g	Wired to portable multichannel DAQ / NR	NR / NR	NR	Forearm sO_2_ monitoring (n = 1)
[34]	Level 3	3 MHz / 1	Pulsed laser; 532 nm; 1.3 ns; up to 2.5 kHz	Watch: 43 × 30 × 24 mm³ / 40 g; backpack: 450 × 300 × 200 mm³ / 7 kg	Wired to body-worn backpack / NR	~8.7 μm / NR	NR	Wrist imaging (n = NR)
[50]	Level 2	9.6 MHz / 64 × 3	OPO laser; 710 and 840 nm; 10 ns; 20 Hz	43 mm × 20 mm / ~20 g	Wired; external passive weight support / NR	~0.2 mm / NR	~5 mm	Brain imaging in mice (n = 3)
[51]	Level 2	Optical fiber sensing / NR	Pulsed laser; 532 and 558 nm	NR / 4.5 g	Optical-fiber and electrical tether / NR	~9.6 μm / NR	~160 μm	Cerebral vascular and metabolic imaging in mice (n = 8)
[111]	Handheld	4 MHz / 256	OPO laser; 680–950 and 1064 nm; 10 Hz	NR / NR	Wired to off-board laser and DAQ / NR	~0.2 mm / NR	Up to 16 mm	Carotid-bifurcation imaging (n = 16)
[112]	Handheld	7 MHz / 128	LED arrays; 850 nm; 70 ns; 4 kHz	NR / NR	Wired to AcousticX system / NR	NR / NR	Up to 34 mm	Wrist vasculature imaging (n = 1)

## 5 Toward fully wearable imaging platforms

Most systems reviewed above remain only partially wearable. The sensing head may be small and light enough to attach to the body, but the power supply, data-acquisition device, excitation laser, data processing, or image display often remain off-board. Handheld or portable systems that are not attached to or carried by the subject are toward-wearable precursors rather than wearable levels. Moving from a wearable probe to a wearable imaging platform requires progress in two directions: tighter system integration and lower hardware burden. Integration brings batteries, control circuits, data acquisition, wireless links, and processing closer to the body. Hardware reduction lowers the number of channels, cables, scan positions, and data rates that the wearable package must support. In the Level 1–4 framework, this transition corresponds to moving from skin-mounted sensing interfaces and partially body-mounted modules toward Level 3 body-worn distributed systems and, ultimately, Level 4 untethered platforms.

### 5.1 System integration and wireless transmission

A practical assessment of full wearability should consider the entire signal chain rather than only the sensing patch. Relevant metrics include device mass and thickness at the skin interface, channel count, frame rate, cable count, wired or wireless operation, battery lifetime, acoustic and optical exposure, data rate, onboard processing latency, wireless bandwidth, reconstruction quality during motion, validation model, number of human participants, and the need for trained operator intervention. Table 1, Table 2 use these fields consistently for one representative system per row and mark unavailable values as NR, making both performance and reporting gaps directly visible.

**Fig. 5:**
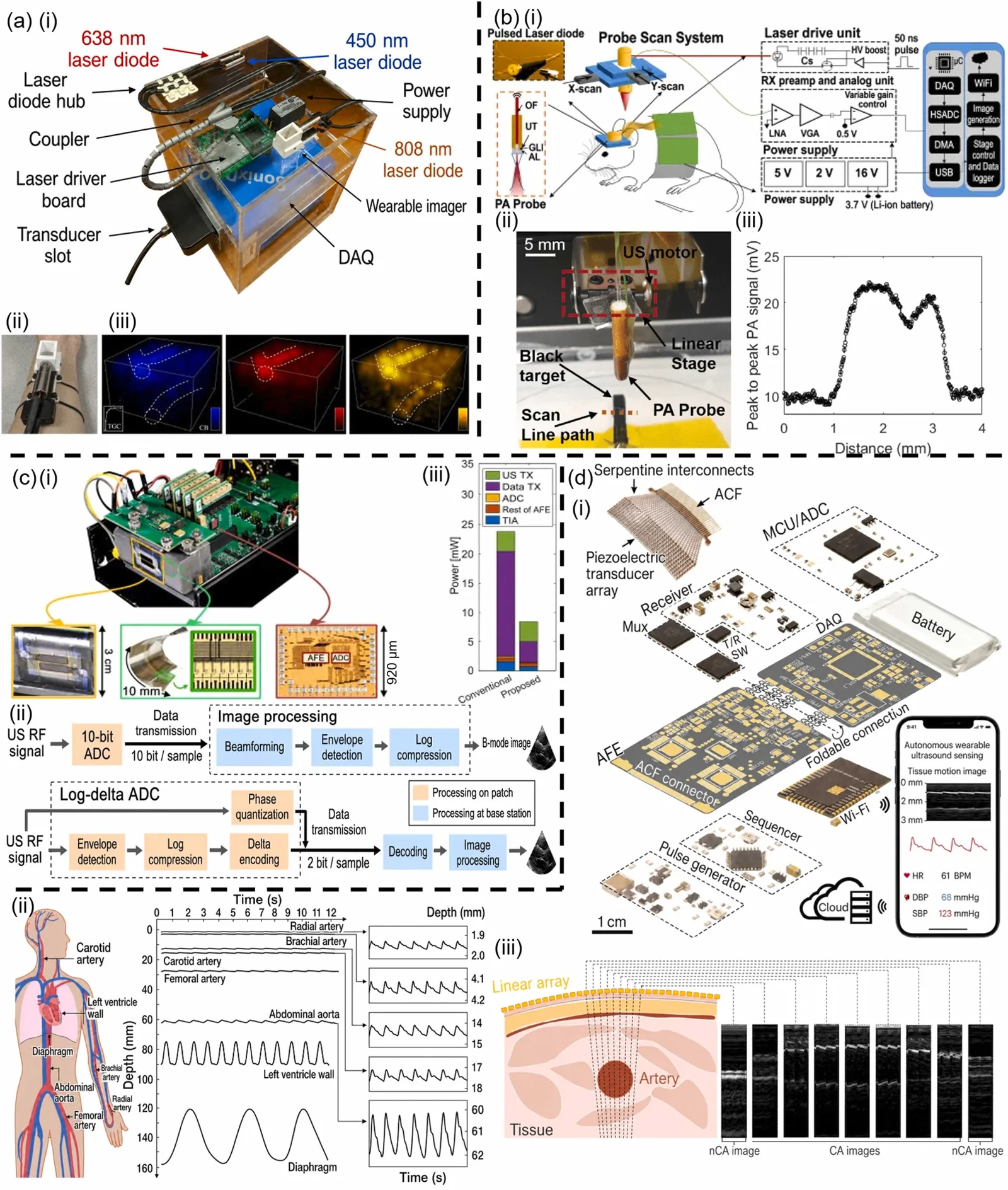
Advances toward fully wearable imaging devices. (a) (i) Photo of the compact photoacoustic imaging system. (ii) Wearable photoacoustic imaging of arm vasculature and (iii) corresponding reconstructed images using 450 nm (left), 638 nm (middle), and 808 nm (right) laser irradiation. (b) (i) A schematic representation of the wearable photoacoustic microscope system. (ii) Experimental photoacoustic imaging of a 1D scan and (iii) the corresponding photoacoustic signal amplitudes. (c) (i) Photo of the integrated ultrasound prototype and close-up photos of the ultrasound transducers (bottom left), 4 × 8-to-1 multiplexer foil (bottom middle), and the silicon die integrating an analog frontend (AFE) and an ADC (bottom right). (ii) Conventional (top) and proposed (bottom) approach for processing ultrasound signals. (iii) Power breakdown comparing the power consumption per channel between a conventional implementation and the proposed techniques. (d) (i) The exploded view of the wireless ultrasound imaging system. (ii) Schematics and measurement results of seven representative dynamic tissue interfaces on the human body. (iii) The cross-sectional view of a soft ultrasound array sensing the carotid artery (left) and the M-mode images of channels with beam penetrating or not penetrating the carotid artery, named as carotid artery (CA) or noncarotid artery (nCA) images (right). Reprinted with permission from [36,37,43,44].

Liu et al. [37] proposed a miniaturized 3D photoacoustic imager based on coherent frequency-domain photoacoustics (Fig. 5**(a)-i**). This system integrated multi-wavelength LD excitation, acoustic detection, driving electronics, and data acquisition into a compact platform with a size of approximately 80 × 25 × 24 mm^3^ and a weight of 21 g. Using 450, 638, and 808 nm LDs, wearable-probe-based functional vascular imaging was demonstrated (Fig. 5**(a)-ii,iii**). Although it was not fully wireless, this work showed that optical excitation, acoustic detection, and backend electronics can be substantially miniaturized for wearable PA monitoring. Dangi et al. [36] further developed a low-cost and portable photoacoustic microscope for point-of-care and wearable applications (Fig. 5**(b)-i**). By integrating the laser source, optical components, power supply, data acquisition, and signal-processing modules into a portable architecture, this system reduced the dependence on benchtop instruments, while a compact probe was used for PA scanning (Fig. 5**(b)-ii,iii**).

For USI, Timmermans et al. [43] proposed a system exploiting transducers and multiplexers on a flexible substrate (Fig. 5**(c)-i**). By integrating flexible transducer arrays with thin-film-transistor multiplexers, this work reduced the required frontend circuitry by 8 times. In addition, the log-delta analog-to-digital converter (ADC) and data compression strategy reduced frontend power consumption by 42% and data transmission by 5 times (Fig. 5**(c)-ii,iii**). These results directly demonstrate the value of frontend integration for wearable ultrasound, where channel count, wiring complexity, power consumption, and data throughput must be minimized. Similarly, a flexible PZT-based row–column addressed 2D PMUT array was proposed by Joshi et al. [113], providing a device-level strategy to reduce interconnection complexity in high-density flexible arrays.

Wireless transmission further enables wearable imaging systems to operate with fewer motion constraints. Lin et al. [44] proposed a fully integrated wearable ultrasound system for monitoring deep tissues in moving subjects (Fig. 5**(d)-i**). This system integrated a flexible ultrasound patch, control electronics, battery power, wireless communication, and machine-learning-assisted signal processing, enabling autonomous monitoring and visualization through a mobile interface (Fig. 5**(d)-ii**). It achieved continuous operation for 12 h and monitored deep-tissue signals at depths up to 164 mm, while motion-mode (M-mode) imaging and physiological motion tracking were demonstrated at different body locations (Fig. 5**(d)-iii**). A portable photoacoustic system featuring wireless data transmission was also reported by Zheng et al. [35], where noninvasive and continuous personal heat strain monitoring was demonstrated. These studies suggest that compact electronics, low-power acquisition, data reduction, wireless communication, and battery-powered operation are key requirements for future fully wearable imaging platforms.

### 5.2 Algorithms, spatial encoding, and quantitative reconstruction

Spatial encoding and compressive acquisition offer one way to reduce the hardware burden. Conventional USI and PAI often rely on dense arrays, many parallel channels, high-speed digitization, and large data throughput. These requirements increase size, power, wiring complexity, and heat. By using known encoding patterns or signal sparsity, compressive methods can reconstruct images from fewer measurements, effectively shifting part of the imaging burden from hardware to computation.

Algorithmic advances are also needed beyond channel reduction. Wearable imaging must operate under deformation, imperfect coupling, motion, changing contact pressure, and variable optical fluence. Therefore, beamforming, image reconstruction, denoising, motion correction, calibration transfer, and quantitative parameter estimation are central to practical deployment. For wearable USI, algorithms must compensate for array deformation and limited aperture while preserving real-time feedback. For wearable PAI, reconstruction must additionally account for wavelength-dependent fluence, acoustic heterogeneity, low pulse energy, and motion between frames or wavelengths.

**Fig. 6:**
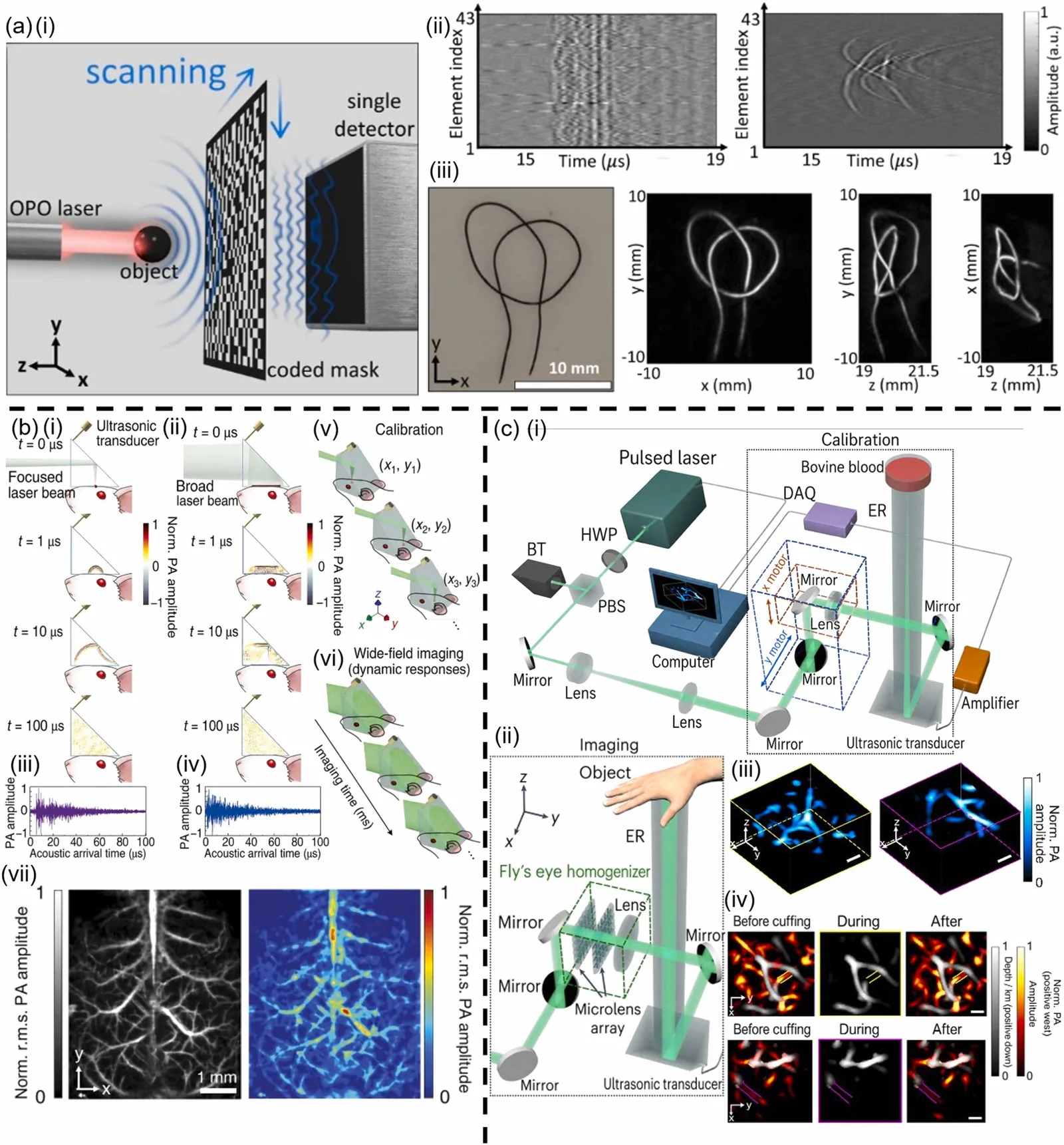
Spatial encoding for single-element photoacoustic imaging. (a) (i) Illustration of the coded aperture with a scanning binary mask. (ii) Original (left) and decoded (right) transducer signals. (iii) Object ground truth image and the maximum amplitude projection (MAP) of the 3D PA image. (b) (i) Simulation of acoustic propagation in the ER in calibration mode. Norm., normalized. (ii) Simulation of acoustic propagation in the ER in wide-field mode. (iii) PA signals detected by a point detector from the simulation in (i). (iv) PA signals detected by a point detector from the simulation in (ii). (v) In calibration mode, light is focused on each pixel to acquire the impulse response encoded by the ER. (vi) Snapshot wide-field imaging where a broad laser beam illuminates the entire FOV to acquire encoded signals. (vii) Calibration image (left) and wide-field image (right) of mouse brain vasculature through an intact skull. (c) (i,ii) Schematic of the system calibration (i) and imaging (ii) procedures. BT, beam trap; DAQ, data acquisition unit; HWP, half-wave plate; PBS, polarizing beam splitter; ER, ergodic relay. The differences between the two modes are highlighted by the black dotted boxes. (iii) 3D PA images of the thenar vasculature of participant 1 (left) and participant 2 (right). (iv) Maximum-amplitude projections of the 3D volumes along the *z*-axis before, during, and after cuffing. The solid lines flank the vessels under investigation. Scale bar: 1 mm. Reprinted with permission from [45,47,114].

One representative approach is coded acoustic aperture imaging. In this strategy, a coded mask or delay-line structure is placed in front of a single-element or low-channel-count transducer, so that acoustic waves from different spatial locations are mixed with distinct temporal or spatial signatures. The received signal can therefore be regarded as a compressed measurement of a virtual transducer array, and the original image can be recovered through inverse reconstruction algorithms. For example, single-detector 3D photoacoustic tomography has been realized by coding one detector (Fig. 6**(a)-i**) into an equivalent 1763-element 2D array [45]. A complex 3D structure produced by black suture was imaged to verify the 3D imaging capabilities (Fig. 6**(a)-ii,iii**). However, practical wearable implementation still faces limitations because some coded aperture systems require large acoustic apertures, mechanical mask rotation, repeated measurements, or careful calibration.

Ergodic relay (ER) imaging further extends the concept of spatial encoding [46,47,[114], [115], [116]]. Instead of using a conventional transducer array, an irregular acoustic relay generates complex and deterministic spatiotemporal interference patterns, allowing a single small transducer to receive encoded wide-field photoacoustic signals. After calibration, each spatial location can be assigned a unique impulse response, enabling snapshot image reconstruction from a single detected waveform (Fig. 6**(b)-i-vi**). Li et al. first demonstrated snapshot wide-field photoacoustic imaging with a single-element detector [47], including *in vivo* mouse-brain functional imaging (Fig. 6**(b)-vii**). More recently, Zhang et al. improved this strategy for object-independent 3D imaging [114]. The system separated calibration and imaging modes using an ER-based optical-acoustic configuration (Fig. 6**(c)-i,ii**), where a single element functioned as 6400 virtual detectors and enabled volumetric hemodynamic imaging at up to 1 kHz. Dynamic 3D vascular responses related to cuffing were further reconstructed (Fig. 6**(c)-iii,iv**), demonstrating the capability of ER-based spatial encoding for high-speed functional PAI. This strategy is particularly promising for wearable PAI because it can greatly reduce probe size and channel count while maintaining wide-field and high-speed imaging capability. Nevertheless, its practical use still depends on calibration stability, motion robustness, low-SNR performance, and real-time reconstruction efficiency.

Beyond the two physical spatial-encoding strategies described above, channel reduction can also be achieved at the acquisition and reconstruction levels. At the frontend, Liao et al. integrated compressive sensing directly into a photoacoustic receiver using matrix-vector-multiplication successive-approximation-register (SAR) ADCs, reducing the output data rate by 4–8 times while retaining information from the full array [117]. Geometry-aware acquisition provides another possibility. For example, ultra-sparse spiral-sampling PAT interlaces detector angles, spatial positions, and illumination wavelengths, achieving reconstruction and spectral-unmixing performance comparable to dense sampling at a sampling ratio as low as 1/30 [118]. Although mechanical scanning is unsuitable for continuously worn devices, similar principles could be implemented through electronically switched subapertures, distributed flexible detectors, or motion-aware sampling.

Complementary reconstruction methods can recover missing spatial information from sparse or limited-view measurements. Physics-based iterative reconstruction with learned regularization [119] and deep algorithm unrolling [120] combines measurement consistency with data-driven image priors, whereas convolutional networks can directly suppress sparse-view and limited-view artifacts [121]. Synthetic-aperture and multiview reconstruction may further compensate for the restricted angular coverage of compact arrays; recent multiview linear-array photoacoustic computed tomography (PACT) combined complementary orientations using iterative deconvolution and improved elevational resolution by up to eightfold [122]. These methods have not all been demonstrated in wearable systems, but they offer promising backend solutions for reducing channel count and reconstructing more complete three-dimensional images. Their practical use will depend on calibration stability, motion robustness, reconstruction latency, and the prevention of unsupported structures introduced by learned priors.

Machine learning may help with adaptive beamforming, low-SNR enhancement, segmentation, physiological parameter extraction, motion classification, and compression of high-rate wearable data streams. Its use should be judged by more than visual image quality. Wearable systems will encounter different subjects, body locations, device placements, skin tones, tissue compositions, and motion states. For clinical or home monitoring, generalization, uncertainty estimation, and validation against accepted reference standards will be essential.

## 6 Translation roadmap and future perspectives

Translation will depend on coordinated progress in hardware, algorithms, and validation. Hardware development should treat soft transducers, multiplexers, compact light sources, drivers, batteries, and thermal management as coupled design variables. Algorithms must handle imperfect coupling, changing geometry, low SNR, and subject motion. Validation must move beyond controlled demonstrations to repeated placement, long monitoring sessions, different users, and comparison with clinical reference measurements.

Safety and usability are not secondary concerns for wearable imaging. Long-duration USI must remain within acoustic-output and heating limits. Wearable PAI must also control optical exposure, wavelength selection, pulse repetition rate, eye safety, and local skin heating. Adhesives and couplants need to preserve acoustic performance without irritation, dehydration, or discomfort. Because many future systems are intended for ambulatory or home use, automatic placement feedback, signal-quality checks, and simple failure detection will be needed.

Safety must be evaluated over the intended monitoring duration. For ultrasound transmission, duty cycle, acoustic output, mechanical index (MI), thermal index (TI), and local temperature rise should be characterized under normal and degraded coupling [30] and assessed against IEC 60601–2–37:2024. For laser-based PAI, maximum permissible exposure (MPE) depends on wavelength, pulse duration, repetition pattern, illuminated area, and total exposure time. For 1–100 ns pulses incident on skin, representative single-pulse radiant-exposure limits are 20 mJ/cm² from 400 to 700 nm; 20 × 10^0.002(λ−700)^ mJ/cm² from 700 to 1050 nm; and 100 mJ/cm² from 1050 to 1400 nm [123]. For pulse trains longer than 10 s, the corresponding average-exposure limits are 200 mW/cm² from 400 to 700 nm, 200 × 10^0.002(λ−700)^ mW/cm² from 700 to 1050 nm, and 1 W/cm² from 1050 to 1400 nm. The most restrictive single-pulse, average-exposure, and multiple-pulse criterion must govern. These values apply to skin under the stated pulse conditions, not to the eye, and each wavelength in a multispectral sequence must be checked individually and over the full pulse train. Repeated excitation and electronics can produce thermal accumulation, so skin-contact temperature should be measured during worst-case continuous operation. Adhesives and couplants should be assessed for irritation, sensitization, dehydration, sweat, pressure, and signal drift. Ambulatory or home-use designs require optical shielding, contact and temperature monitoring, placement and signal-quality checks, and automatic shutdown for detachment, degraded coupling, blocked optical output, battery faults, or corrupted data.

Table 3 consolidates these translational barriers and links each one to a practical engineering direction and the validation evidence still required. Its main implication is that progress toward Level 4 wearability must be demonstrated through long-duration safety, motion, battery, and human-validation benchmarks rather than device miniaturization alone.

**Table 3:** Key barriers and future directions for translational wearable PA/US imaging.

**Barrier**	**Why it matters**	**Promising directions**	**Evidence needed**
Stable skin coupling	Longitudinal imaging depends on reproducible acoustic contact during motion and sweating	Bioadhesive couplants, anti-dehydration gels, conformal mechanics, automatic coupling-quality feedback	Multi-hour and multi-day human studies across body locations and motion states
Compact light delivery for PAI	PA signal strength and quantitative accuracy depend on safe and efficient optical excitation	VCSEL/LD arrays, integrated diffusers, wavelength multiplexing, thermal-aware packaging	*In vivo* fluence, temperature, SNR, and safety measurements
Low-power electronics and wireless data	Untethered operation is limited by channel count, acquisition rate, battery capacity, and heat	application-specific integrated circuit (ASIC) front ends, multiplexing, row-column addressing, compressed acquisition, onboard reconstruction	Battery lifetime, latency, data-rate, and thermal benchmarks during continuous use
Motion-robust reconstruction	Wearable systems must image during natural movement and variable placement	Deformation-aware beamforming, sensor fusion, motion correction, calibration transfer, uncertainty estimation	Validation during walking, exercise, posture changes, and repeated donning
Clinical validation and regulation	Adoption requires reliable measurements linked to clinical decisions	Task-specific endpoints, comparison with standard-of-care imaging, automated quality control	Prospective studies with diverse participants and clinically meaningful performance metrics
Cumulative acoustic, optical, thermal, and skin-contact safety	Repeated excitation, electronics heating, adhesives, and couplants can create risks not captured by short laboratory measurements	IEC 60601–2–37 acoustic-output assessment; ANSI Z136.1/Z136.3 laser exposure assessment; IEC 62471 non-laser source assessment; exposure-aware control, thermal sensing, optical shielding, contact monitoring, and automatic shutdown	Worst-case multi-hour tests of MI/TI and acoustic output, wavelength- and pulse-train-dependent optical exposure, device and skin temperature, biocompatibility, skin response, and fault conditions

## 7 Conclusion and discussion

In summary, wearable USI and PAI have progressed from isolated probes toward more integrated body-conformal imaging systems. Wearable USI is currently more mature, with bioadhesive probes, stretchable arrays, and application-specific patches demonstrated for cardiovascular, cerebral, bladder, breast, and other targets. Wearable PAI is earlier in its development but is moving from portable laser-diode systems toward soft patches, watch-type modules, PMUT/VCSEL sensing heads, and head-mounted preclinical imagers. The most compelling future systems will likely combine the strengths of both modalities: ultrasound anatomy and motion together with photoacoustic hemodynamics, oxygenation, temperature, and molecular contrast.

Despite this progress, important limitations remain. Many current systems are still only partially wearable because they require external power supplies, data-acquisition units, laser drivers, cables, off-board computation, or trained operators. For wearable USI, unstable coupling, array deformation, limited aperture size, and tissue motion can degrade beamforming accuracy and image quality. For wearable PAI, low optical pulse energy from miniaturized light sources, wavelength-dependent fluence, thermal safety, calibration drift, and relatively low signal-to-noise ratio remain major barriers to deep tissue imaging and quantitative imaging. In addition, most reported systems have been validated in controlled laboratory settings or small cohorts, and their long-term performance during daily activity, sweating, repeated placement, and diverse body types remains insufficiently studied.

Future PA/US wearables will therefore require application-specific designs that combine soft transducers, compact light sources, low-power electronics, wireless communication, battery and thermal management, robust coupling, real-time reconstruction, and quantitative algorithms. Advanced algorithms, especially the emerging machine-learning-based approaches, which compensate for device deformation, imperfect contact, motion, and unknown optical fluence, will be essential. Standardized reporting of device mass, thickness, cable count, battery life, acoustic and optical exposure, data rate, latency, wireless bandwidth, and image quality during motion would also make it easier to compare systems across studies.

Looking forward, wearable PA/US imaging could enable several important applications. In cardiovascular care, these systems may support continuous monitoring of vessel diameter, pulse-wave dynamics, cardiac motion, blood oxygenation, and cuff-free blood pressure. In neurovascular monitoring, they may provide a wearable assessment of cerebral blood flow and oxygenation. In rehabilitation and sports medicine, combined US structural imaging and PA perfusion mapping could help evaluate muscle function, inflammation, and recovery. Other promising directions include bladder monitoring, breast cancer treatment monitoring, wound healing assessment, peripheral vascular disease monitoring, and home-based longitudinal health tracking. If the remaining challenges in integration, safety, quantification, and human validation are addressed, wearable PA/US imaging could become a useful tool for continuous monitoring, early disease detection, rehabilitation, neuroscience, and personalized care.

## CRediT authorship contribution statement

**Mingze Luo:** Writing – review & editing, Writing – original draft, Visualization, Investigation, Data curation, Conceptualization. **Shunyao Zhang:** Writing – review & editing, Writing – original draft, Visualization, Investigation. **Wentao Jiang:** Writing – review & editing, Writing – original draft, Visualization, Investigation. **Jingyi Miao:** Writing – review & editing, Writing – original draft, Investigation. **Zihan Zhao:** Writing – review & editing, Writing – original draft, Investigation. **Lei S. Li:** Writing – review & editing, Supervision, Project administration, Funding acquisition, Conceptualization.

## Declaration of Competing Interest

L.S.L. has a financial interest in BLOCH Quantum Imaging Solutions, although they did not support this work. The other authors declare no competing financial interests.

## Data Availability

No data was used for the research described in the article.

## References

[1] Weissleder R., Nahrendorf M. (2015). Advancing biomedical imaging. Proc. Natl. Acad. Sci..

[2] Tempany C.M., McNeil B.J. (2001). Advances in biomedical imaging. Jama.

[3] Ntziachristos V., Ripoll J., Wang L.V., Weissleder R. (2005). Looking and listening to light: the evolution of whole-body photonic imaging. Nat. Biotechnol..

[4] Li L., Wang L.V. (2021). Recent advances in photoacoustic tomography. BME Front. 2021.

[5] Huang Q., Zeng Z. (2017). A review on real-time 3D ultrasound imaging technology. BioMed. Res. Int..

[6] Jensen J.A. (2007). Medical ultrasound imaging. Progress. Biophys. Mol. Biol..

[7] Wells P.N. (2006). Ultrasound imaging. Phys. Med. & Biol..

[8] Wang L.V., Yao J. (2016). A practical guide to photoacoustic tomography in the life sciences. Nat. Methods.

[9] Yao J., Wang L., Yang J.-M., Maslov K.I., Wong T.T., Li L., Huang C.-H., Zou J., Wang L.V. (2015). High-speed label-free functional photoacoustic microscopy of mouse brain in action. Nat. Methods.

[10] Li L., Yao J., Wang L.V. (2016). Wiley Encyclopedia of Electrical and Electronics Engineering.

[11] Yao J., Kaberniuk A.A., Li L., Shcherbakova D.M., Zhang R., Wang L., Li G., Verkhusha V.V., Wang L.V. (2016). Multiscale photoacoustic tomography using reversibly switchable bacterial phytochrome as a near-infrared photochromic probe. Nat. Methods.

[12] Zhou M., Li L., Yao J., Bouchard R.R., Wang L.V., Li C. (2017). Design and Applications of Nanoparticles in Biomedical Imaging.

[13] Li L., Shemetov A.A., Baloban M., Hu P., Zhu L., Shcherbakova D.M., Zhang R., Shi J., Yao J., Wang L.V. (2018). Small near-infrared photochromic protein for photoacoustic multi-contrast imaging and detection of protein interactions in vivo. Nat. Commun..

[14] Lin L., Hu P., Shi J., Appleton C.M., Maslov K., Li L., Zhang R., Wang L.V. (2018). Single-breath-hold photoacoustic computed tomography of the breast. Nat. Commun..

[15] Shi J., Wong T.T.W., He Y., Li L., Zhang R., Yung C.S., Hwang J., Maslov K., Wang L.V. (2019). High-resolution, high-contrast mid-infrared imaging of fresh biological samples with ultraviolet-localized photoacoustic microscopy. Nat. Photonics.

[16] Wu Z., Li L., Yang Y., Hu P., Li Y., Yang S.-Y., Wang L.V., Gao W. (2019). A microrobotic system guided by photoacoustic computed tomography for targeted navigation in intestines in vivo. Sci. Robot..

[17] Li L., Hsu H.-C., Verkhusha V.V., Wang L.V., Shcherbakova D.M. (2021). Multiscale photoacoustic tomography of a genetically encoded near-infrared FRET biosensor. Adv. Sci..

[18] Li L., Patil D., Petruncio G., Harnden K.K., Somasekharan J.V., Paige M., Wang L.V., Salvador-Morales C. (2021). Integration of multitargeted polymer-based contrast agents with photoacoustic computed tomography: an imaging technique to visualize breast cancer intratumor heterogeneity. ACS Nano.

[19] Zhang Y.S., Yao J., Zhang C., Li L., Wang L.V., Xia Y. (2014). Optical-resolution photoacoustic microscopy for volumetric and spectral analysis of histological and immunochemical samples. Angew. Chem. Int. Ed..

[20] Gu M. (2000).

[21] Luker G.D., Luker K.E. (2008). Optical imaging: current applications and future directions. J. Nucl. Med..

[22] Li L., Zhu L., Ma C., Lin L., Yao J., Wang L., Maslov K., Zhang R., Chen W., Shi J., Wang L.V. (2017). Single-impulse panoramic photoacoustic computed tomography of small-animal whole-body dynamics at high spatiotemporal resolution. Nat. Biomed. Eng..

[23] Menozzi L., Yao J. (2024). Deep tissue photoacoustic imaging with light and sound. npj Imaging.

[24] Park B., Oh D., Kim J., Kim C. (2023). Functional photoacoustic imaging: from nano-and micro-to macro-scale. Nano Converg..

[25] Zhang H.F., Maslov K., Stoica G., Wang L.V. (2006). Functional photoacoustic microscopy for high-resolution and noninvasive in vivo imaging. Nat. Biotechnol..

[26] Liu C., Wang L. (2022). Functional photoacoustic microscopy of hemodynamics: a review. Biomed. Eng. Lett..

[27] Li L., Yeh C., Hu S., Wang L., Soetikno B.T., Chen R., Zhou Q., Shung K.K., Maslov K.I., Wang L.V. (2014). Fully motorized optical-resolution photoacoustic microscopy. Opt. Lett..

[28] Gao X., Chen X., Hu H., Wang X., Yue W., Mu J., Lou Z., Zhang R., Shi K., Chen X. (2022). A photoacoustic patch for three-dimensional imaging of hemoglobin and core temperature. Nat. Commun..

[29] Huang H., Wu R.S., Lin M., Xu S. (2023). Emerging wearable ultrasound technology. IEEE Trans. Ultrason. Ferroelectr. Freq. Control..

[30] Song P., Andre M., Chitnis P., Xu S., Croy T., Wear K., Sikdar S. (2023). Clinical, safety, and engineering perspectives on wearable ultrasound technology: a review. IEEE Trans. Ultrason. Ferroelectr. Freq. Control..

[31] Zhou S., Park G., Lin M., Yang X., Xu S. (2025). Wearable ultrasound technology. Nat. Rev. Bioeng..

[32] Zhu Y., Xu G., Yuan J., Jo J., Gandikota G., Demirci H., Agano T., Sato N., Shigeta Y., Wang X. (2018). Light emitting diodes based photoacoustic imaging and potential clinical applications. Sci. Rep..

[33] Xia W., Kuniyil Ajith Singh M., Maneas E., Sato N., Shigeta Y., Agano T., Ourselin S., J. West S., E. Desjardins A. (2018). Handheld real-time LED-based photoacoustic and ultrasound imaging system for accurate visualization of clinical metal needles and superficial vasculature to guide minimally invasive procedures. Sensors.

[34] Zhang T., Guo H., Qi W., Xi L. (2024). Wearable photoacoustic watch for humans. Opt. Lett..

[35] Zheng Z., Jin H., Wang X., Zheng Y. (2024). 2024 IEEE Biomedical Circuits and Systems Conference (BioCAS).

[36] Dangi A., Agrawal S., Datta G.R., Srinivasan V., Kothapalli S.-R. (2019). Towards a low-cost and portable photoacoustic microscope for point-of-care and wearable applications. IEEE Sens. J..

[37] Liu S., Tang K., Feng X., Jin H., Gao F., Zheng Y. (2019). Toward wearable healthcare: a miniaturized 3d imager with coherent frequency-domain photoacoustics. IEEE Trans. Biomed. Circuits Syst..

[38] Moisello E., Novaresi L., Sarkar E., Malcovati P., Costa T.L., Bonizzoni E. (2024). PMUT and CMUT devices for biomedical applications: a review. Ieee Access..

[39] Wang C., Chen X., Wang L., Makihata M., Liu H.-C., Zhou T., Zhao X. (2022). Bioadhesive ultrasound for long-term continuous imaging of diverse organs. Science.

[40] Hu H., Zhu X., Wang C., Zhang L., Li X., Lee S., Huang Z., Chen R., Chen Z., Wang C. (2018). Stretchable ultrasonic transducer arrays for three-dimensional imaging on complex surfaces. Sci. Adv..

[41] Zeng L., Liu G., Yang D., Ji X. (2013). Portable optical-resolution photoacoustic microscopy with a pulsed laser diode excitation. Appl. Phys. Lett..

[42] M. Shao, Z. Lu, S. Liu, N. Ruiter, C. Klahn, Wearable photoacoustic LED array based on frequency-modulated excitation and 3D-printed microlenses, Proc. SPIE 14098, Tissue Optics and Photonics IV (2026) 1409827.

[43] Timmermans M., van Oosterhout K., Fattori M., van Neer P., Harpe P., Cantatore E. (2025). An ultrasound imaging system exploiting transducers and multiplexers on a flexible substrate together with a log-delta CMOS ADC. npj Flex. Electron..

[44] Lin M., Zhang Z., Gao X., Bian Y., Wu R.S., Park G., Lou Z., Zhang Z., Xu X., Chen X. (2024). A fully integrated wearable ultrasound system to monitor deep tissues in moving subjects. Nat. Biotechnol..

[45] Hahamovich E., Monin S., Levi A., Hazan Y., Rosenthal A. (2022). Single-detector 3D optoacoustic tomography via coded spatial acoustic modulation. Commun. Eng..

[46] Li L., Li Y., Zhang Y., Wang L.V. (2021). Snapshot photoacoustic topography through an ergodic relay of optical absorption in vivo. Nat. Protoc..

[47] Li Y., Li L., Zhu L., Maslov K., Shi J., Hu P., Bo E., Yao J., Liang J., Wang L. (2020). Snapshot photoacoustic topography through an ergodic relay for high-throughput imaging of optical absorption. Nat. Photonics.

[48] Wang C., Qi B., Lin M., Zhang Z., Makihata M., Liu B., Zhou S., Huang Y.-h, Hu H., Gu Y. (2021). Continuous monitoring of deep-tissue haemodynamics with stretchable ultrasonic phased arrays. Nat. Biomed. Eng..

[49] Zhou S., Gao X., Park G., Yang X., Qi B., Lin M., Huang H., Bian Y., Hu H., Chen X. (2024). Transcranial volumetric imaging using a conformal ultrasound patch. Nature.

[50] Tang J., Coleman J.E., Dai X., Jiang H. (2016). Wearable 3-D photoacoustic tomography for functional brain imaging in behaving rats. Sci. Rep..

[51] Zhong X., Liang Y., Wang X., Lan H., Bai X., Jin L., Guan B.-O. (2024). Free-moving-state microscopic imaging of cerebral oxygenation and hemodynamics with a photoacoustic fiberscope. Light Science & Applications.

[52] Liang X., Guo H., Li L., Li T., Li B., Qin W., Zhao Y., Xi L., Yuan Z. (2025). Wearable photoacoustic microscopy for ultrafast stroke metabolic imaging in freely moving mice. Cell. Rep. Phys. Sci..

[53] Liu H.-C., Zeng Y., Gong C., Chen X., Kijanka P., Zhang J., Genyk Y., Tchelepi H., Wang C., Zhou Q. (2024). Wearable bioadhesive ultrasound shear wave elastography. Sci. Adv..

[54] Duque Londono C., Cones S.F., Deng J., Wu J., Yuk H., Guza D.E., Mooney T.A., Zhao X. (2024). Bioadhesive interface for marine sensors on diverse soft fragile species. Nat. Commun..

[55] Singh M., Teodorescu D.L., Rowlett M., Wang S.X., Balcells M., Park C., Bernardo B., McGarel S., Reeves C., Mehra M.R. (2024). A tunable soft silicone bioadhesive for secure anchoring of diverse medical devices to wet biological tissue. Adv. Mater..

[56] Wu J., Deng J., Theocharidis G., Sarrafian T.L., Griffiths L.G., Bronson R.T., Veves A., Chen J., Yuk H., Zhao X. (2024). Adhesive anti-fibrotic interfaces on diverse organs. Nature.

[57] Hu H., Huang H., Li M., Gao X., Yin L., Qi R., Wu R.S., Chen X., Ma Y., Shi K. (2023). A wearable cardiac ultrasound imager. Nature.

[58] Harvey G., Gachagan A., Mackersie J.W., McCunnie T., Banks R. (2009). Flexible ultrasonic transducers incorporating piezoelectric fibres. IEEE Trans. Ultrason. Ferroelectr. Freq. Control..

[59] Powell D., Hayward G. (1996). Flexible ultrasonic transducer arrays for nondestructive evaluation applications. II. Performance assessment of different array configurations. IEEE Trans. Ultrason. Ferroelectr. Freq. Control..

[60] Qi Y., Jafferis N.T., Lyons K., Jr, Lee C.M., Ahmad H., McAlpine M.C. (2010). Piezoelectric ribbons printed onto rubber for flexible energy conversion. Nano Lett..

[61] Wang Z., Xue Q.-T., Chen Y.-Q., Shu Y., Tian H., Yang Y., Xie D., Luo J.-W., Ren T.-L. (2015). A flexible ultrasound transducer array with micro-machined bulk PZT. Sensors.

[62] Zhuang X., Lin D.-S., Oralkan Ö., Khuri-Yakub B.T. (2008). Fabrication of flexible transducer arrays with through-wafer electrical interconnects based on trench refilling with PDMS. J. Micro Syst..

[63] Qiu Y., Gigliotti J.V., Wallace M., Griggio F., Demore C.E., Cochran S., Trolier-McKinstry S. (2015). Piezoelectric micromachined ultrasound transducer (PMUT) arrays for integrated sensing, actuation and imaging. Sensors.

[64] Jung J., Lee W., Kang W., Shin E., Ryu J., Choi H. (2017). Review of piezoelectric micromachined ultrasonic transducers and their applications. J. Micromech. Microeng..

[65] Omidvar A., Rohling R.N., Cretu E., Cresswell M.E., Hodgson A.J. (2024). Preliminary demonstration of pulse-echo imaging with a long monolithic flexible CMUT array. IEEE Open. J. Ultrason. Ferroelectr. Freq. Control..

[66] Lee S.-M., Liang X., Jo Y., Lee T., Bang S., Oh C., Kwon Y.S., Jung J., Lee B.C., Lee H.J. (2025). Flexible ultrasound transducer array with statically adjustable curvature for anti-inflammatory treatment. npj Flex. Electron..

[67] Ma Y., Hu X., Gong Y., Xu X., Pang W., Niu P., Wang Z. (2025). 2025 IEEE International Ultrasonics Symposium (IUS).

[68] Gami P., Roy T., Liang P., Kemper P., Travagliati M., Baldasarre L., Bart S., Konofagou E.E. (2025). vivo characterization of central arterial properties using a miniaturized pMUT array compared to a clinical transducer: a feasibility study towards wearable pulse wave imaging. IEEE Trans. Biomed. Eng..

[69] Wei W., Gong Y., Xu X., Pang W., Wang Z., Ma Y., Niu P. (2026). Development of high frequency and wideband PMUTs toward skincare assessment applications. J. Micro Syst..

[70] Caronti A., Caliano G., Carotenuto R., Savoia A., Pappalardo M., Cianci E., Foglietti V. (2006). Capacitive micromachined ultrasonic transducer (CMUT) arrays for medical imaging. Microelectron. J..

[71] Roy K., Lee J.E.-Y., Lee C. (2023). Thin-film PMUTs: a review of over 40 years of research. Microsyst. & Nanoeng..

[72] Wong S.J., Roy K., Lee C., Zhu Y. (2024). Thin-film piezoelectric micromachined ultrasound transducers in biomedical applications: a review. IEEE Trans. Ultrason. Ferroelectr. Freq. Control..

[73] Chen J., Liu J., Chen W., Shang D., Zhang Q., Li Y., Zheng H., Gu D., Wu D., Ma T. (2024). Skin-conformable flexible and stretchable ultrasound transducer for wearable imaging. IEEE Trans. Ultrason. Ferroelectr. Freq. Control..

[74] Kim T., Cui Z., Chang W.-Y., Kim H., Zhu Y., Jiang X. (2019). Flexible 1–3 composite ultrasound transducers with silver-nanowire-based stretchable electrodes. IEEE Trans. Ind. Electron..

[75] Lin Y., Li Q., Ding C., Wang J., Yuan W., Liu Z., Su W., Cui Z. (2022). High-resolution and large-size stretchable electrodes based on patterned silver nanowires composites. Nano Res..

[76] Pietrangelo S.J., Lee H.-S., Sodini C.G. (2018). Intracranial Pressure & Neuromonitoring XVI.

[77] Zhang L., Marcus C., Lin D., Mejorado D., Schoen S.J., Jr, Pierce T.T., Kumar V., Fernandez S.V., Hunt D., Li Q. (2024). A conformable phased-array ultrasound patch for bladder volume monitoring. Nat. Electron..

[78] Pu C., Fu B., Guo L., Xu H., Peng C. (2024). A stretchable and wearable ultrasonic transducer array for bladder volume monitoring application. IEEE Sens. J..

[79] Du W., Zhang L., Suh E., Lin D., Marcus C., Ozkan L., Ahuja A., Fernandez S., Shuvo I.I., Sadat D. (2023). Conformable ultrasound breast patch for deep tissue scanning and imaging. Sci. Adv..

[80] Kang D.-H., Cho S., Kim H.Y., Shim S., Kim D.H., Jeong B., Lee Y.S., Park E.-A., Lee W., Kim H. (2025). Silicon nanocolumn-based disposable and flexible ultrasound patches. Nat. Commun..

[81] Xu X., Yang W., Wang Z., Ma Y., Gong Y., Wang Z., Jian X., Wang X., Zhou S., Pang W. (2026). High-uniformity miniaturized PMUT array with broadband and high-sensitivity for wearable ultrasound imaging. Microsyst. & Nanoeng..

[82] Petermann K. (1991).

[83] Sciamanna M., Shore K.A. (2015). Physics and applications of laser diode chaos. Nat. Photonics.

[84] Zeng L., Liu G., Yang D., Ji X. (2012). 3D-visual laser-diode-based photoacoustic imaging. Opt. Express.

[85] Daoudi K., Van Den Berg P., Rabot O., Kohl A., Tisserand S., Brands P., Steenbergen W. (2014). Handheld probe integrating laser diode and ultrasound transducer array for ultrasound/photoacoustic dual modality imaging. Opt. Express.

[86] Erfanzadeh M., Salehi H.S., Kumavor P., Zhu Q. (2016). Photons Plus Ultrasound: Imaging and Sensing 2016.

[87] Deng L., Chen Q., Bai Y., Liu G., Zeng L., Ji X. (2021). Compact long-working-distance laser-diode-based photoacoustic microscopy with a reflective objective. Chin. Opt. Lett..

[88] Li X., Tsang V.T., Kang L., Zhang Y., Wong T.T. (2021). High-speed high-resolution laser diode-based photoacoustic microscopy for in vivo microvasculature imaging. Vis. Comput. Ind. Biomed. Art..

[89] Ledentsov N.N., Makarov O.Y., Shchukin V.A., Kalosha V., Ledentsov N., Chrochos L., Sanayeh M.B., Turkiewicz J.P. (2022). High speed VCSEL technology and applications. J. Light. Technol..

[90] Michalzik R. (2012). fundamentals, technology and applications of vertical-cavity surface-emitting lasers.

[91] Cho S., Kim M., Ahn J., Kim Y., Lim J., Park J., Kim H.H., Kim W.J., Kim C. (2024). An ultrasensitive and broadband transparent ultrasound transducer for ultrasound and photoacoustic imaging in-vivo. Nat. Commun..

[92] Park J., Park B., Kim T.Y., Jung S., Choi W.J., Ahn J., Yoon D.H., Kim J., Jeon S., Lee D. (2021). Quadruple ultrasound, photoacoustic, optical coherence, and fluorescence fusion imaging with a transparent ultrasound transducer. Proc. Natl. Acad. Sci..

[93] Panchawagh H.V., Agrawal S., Soukup B.H., Djordjev K. (2025).

[94] Fang Y., Zhao L., Zhang H., Lu Y. (2024). 2024 IEEE Ultrasonics, Ferroelectrics, and Frequency Control Joint Symposium (UFFC-JS).

[95] Hariri A., Lemaster J., Wang J., Jeevarathinam A.S., Chao D.L., Jokerst J.V. (2018). The characterization of an economic and portable LED-based photoacoustic imaging system to facilitate molecular imaging. Photoacoustics.

[96] Zhu Y., Feng T., Cheng Q., Wang X., Du S., Sato N., Yuan J., Kuniyil Ajith Singh M. (2020). Towards clinical translation of LED-based photoacoustic imaging: a review. Sensors.

[97] Joseph Francis K., Boink Y.E., Dantuma M., Ajith Singh M.K., Manohar S., Steenbergen W. (2020). Tomographic imaging with an ultrasound and LED-based photoacoustic system. Biomed. Opt. Express.

[98] Dai X., Yang H., Jiang H. (2017). vivo photoacoustic imaging of vasculature with a low-cost miniature light emitting diode excitation. Opt. Lett..

[99] Bulsink R., Kuniyil Ajith Singh M., Xavierselvan M., Mallidi S., Steenbergen W., Francis K.J. (2021). Oxygen saturation imaging using LED-based photoacoustic system. Sensors.

[100] Van Heumen S., Riksen J.J., Singh M.K.A., Van Soest G., Vasilic D. (2023). LED-based photoacoustic imaging for preoperative visualization of lymphatic vessels in patients with secondary limb lymphedema. Photoacoustics.

[101] Dangi A., Cheng C.Y., Agrawal S., Tiwari S., Datta G.R., Benoit R.R., Pratap R., Trolier-Mckinstry S., Kothapalli S.-R. (2019). A photoacoustic imaging device using piezoelectric micromachined ultrasound transducers (PMUTs). IEEE Trans. Ultrason. Ferroelectr. Freq. Control..

[102] Fang Y., Bao A., Shi Z., Gan J., Sheng B., Zhang H., Lu Y. (2025). A miniature photoacoustic sensing system with advanced PMUT and VCSEL devices. IEEE Trans. Ultrason. Ferroelectr. Freq. Control..

[103] Chen H., Mirg S., Osman M., Agrawal S., Cai J., Biskowitz R., Minotto J., Kothapalli S.-R. (2021). A high sensitivity transparent ultrasound transducer based on PMN-PT for ultrasound and photoacoustic imaging. IEEE Sens. Lett..

[104] Chen R., He Y., Shi J., Yung C., Hwang J., Wang L.V., Zhou Q. (2020). Transparent high-frequency ultrasonic transducer for photoacoustic microscopy application. IEEE Trans. Ultrason. Ferroelectr. Freq. Control..

[105] Dangi A., Agrawal S., Kothapalli S.-R. (2019). Lithium niobate-based transparent ultrasound transducers for photoacoustic imaging. Opt. Lett..

[106] Chen H., Agrawal S., Dangi A., Wible C., Osman M., Abune L., Jia H., Rossi R., Wang Y., Kothapalli S.-R. (2019). Optical-resolution photoacoustic microscopy using transparent ultrasound transducer. Sensors.

[107] Chen H., Agrawal S., Osman M., Minotto J., Mirg S., Liu J., Dangi A., Tran Q., Jackson T., Kothapalli S.-R. (2022). A transparent ultrasound array for real-time optical, ultrasound, and photoacoustic imaging. BME Front. 2022.

[108] Ilkhechi A.K., Ceroici C., Li Z., Zemp R. (2020). Transparent capacitive micromachined ultrasonic transducer (CMUT) arrays for real-time photoacoustic applications. Opt. Express.

[109] Park J., Park B., Ahn J., Kim D., Kim J.Y., Kim H.H., Kim C. (2022). Opto-ultrasound biosensor for wearable and mobile devices: realization with a transparent ultrasound transducer. Biomed. Opt. Express.

[110] Bing R.W., Shijo V., Zheng E., Zheng W., Huang C., Xia J. (2023). 2023 IEEE International Ultrasonics Symposium (IUS).

[111] Ivankovic I., Merčep E., Schmedt C.-G., Dean-Ben X.L., Razansky D. (2019). Real-time volumetric assessment of the human carotid artery: handheld multispectral optoacoustic tomography. Radiology.

[112] Agrawal S., Kuniyil Ajith Singh M., Johnstonbaugh K., Han D.C., Pameijer C.R., Kothapalli S.-R. (2021). Photoacoustic imaging of human vasculature using LED versus laser illumination: a comparison study on tissue phantoms and in vivo humans. Sensors.

[113] Joshi S.V., Sadeghpour S., Kraft M. (2024). Flexible PZT-based row-column addressed 2-D PMUT array. IEEE Trans. Ultrason. Ferroelectr. Freq. Control..

[114] Zhang Y., Hu P., Li L., Cao R., Khadria A., Maslov K., Tong X., Zeng Y., Jiang L., Zhou Q. (2024). Ultrafast longitudinal imaging of haemodynamics via single-shot volumetric photoacoustic tomography with a single-element detector. Nat. Biomed. Eng..

[115] Li Y., Li L., Zhu L., Shi J., Maslov K., Wang L.V. (2020). Photoacoustic topography through an ergodic relay for functional imaging and biometric application in vivo. J. Biomed. Opt..

[116] Li Y., Wong T.T., Shi J., Hsu H.-C., Wang L.V. (2020). Multifocal photoacoustic microscopy using a single-element ultrasonic transducer through an ergodic relay. Light Science & Applications.

[117] Liao H.-C., Zhang S., Su Y., Govinday A., Zou Y., Wang W., Boominathan V., Veeraraghavan A., Li L.S., Yang K. (2025). Compressive sensing photoacoustic imaging receiver with matrix-vector-multiplication SAR ADC. IEEE J. Solid. -State Circuits.

[118] Zhong Y., Zhang X., Mo Z., Zhang S., Nie L., Chen W., Qi L. (2024). Spiral scanning and self-supervised image reconstruction enable ultra-sparse sampling multispectral photoacoustic tomography. Photoacoustics.

[119] Wang T., He M., Shen K., Liu W., Tian C. (2022). Learned regularization for image reconstruction in sparse-view photoacoustic tomography. Biomed. Opt. Express.

[120] Ge J., Mo Z., Zhang S., Zhang X., Zhong Y., Liang Z., Hu C., Chen W., Qi L. (2024). Image reconstruction of multispectral sparse sampling photoacoustic tomography based on deep algorithm unrolling. Photoacoustics.

[121] Davoudi N., Deán-Ben X.L., Razansky D. (2019). Deep learning optoacoustic tomography with sparse data. Nat. Mach. Intell..

[122] Miao J., Zhang S., Sun G., Li L. (2025). Multiview linear-array photoacoustic computed tomography with improved elevational resolution in 3D. Biomed. Opt. Express.

[123] Institute A.N.S. (2022).

